# A Text-Guided Lesion Mining Vision–Language Ordinal Classification Framework for Diabetic Retinopathy Grading

**DOI:** 10.3390/jimaging12090412

**Published:** 2026-09-01

**Authors:** Jiawen Huang, Jinxia Shang

**Affiliations:** 1 Faculty of Information Engineering and Automation, Kunming University of Science and Technology, Kunming 650500, China; hjw@stu.kust.edu.cn; 2 Yunnan Key Laboratory of Computer Technology Applications, Kunming University of Science and Technology, Kunming 650500, China

**Keywords:** diabetic retinopathy, vision–language model, cross-layer lesion mining, ordinal distribution alignment, multi-center feature constraint

## Abstract

Diabetic retinopathy (DR) is a major retinal disease that can cause visual impairment and irreversible blindness. Accurate automated DR grading is essential for large-scale screening and timely clinical intervention. However, most existing methods rely primarily on visual features for classification. Moreover, they often overlook the ordinal structure of DR severity and the intra-class phenotypic heterogeneity arising from diverse lesion combinations. To address these issues, based on the semantic prior information provided by RetiZero, we propose a text-guided lesion mining vision–language ordinal classification framework for DR grading. The proposed framework introduces a text-guided cross-layer lesion mining module that exploits semantic response differences between normal-tissue and lesion-related textual prompts, thereby guiding multi-level visual patch features toward lesion regions relevant to DR grading. To explicitly model the ordered progression of DR severity, we design a conditional ordinal regression branch and an ordinal distribution alignment strategy that jointly encourage the predictions to follow the inherent order of DR grades. Moreover, we introduce a multi-center feature constraint to capture diverse intra-grade phenotypic patterns and enhance feature discriminability. Experiments on APTOS 2019 show that the proposed method achieves 86.3% accuracy, 90.6% AUC, and 70.9% Macro-F1, which improved by 2.4, 0.7, and 5.5 percentage points compared to RetiZero. Furthermore, under the standardized leave-one-domain-out protocol of GDRNet, the proposed method achieves the highest reported average accuracy of 58.6% across six public DR datasets, exceeding the reported result of PAF (54.6%) by 4.0 percentage points. Nevertheless, our approach is limited in F1 and AUC metrics, for which GDRNet delivers superior performance. These results suggest that the proposed framework can improve DR grading performance and the cross-dataset generalization ability of the model to a certain extent.

## 1. Introduction

**Figure 1:**
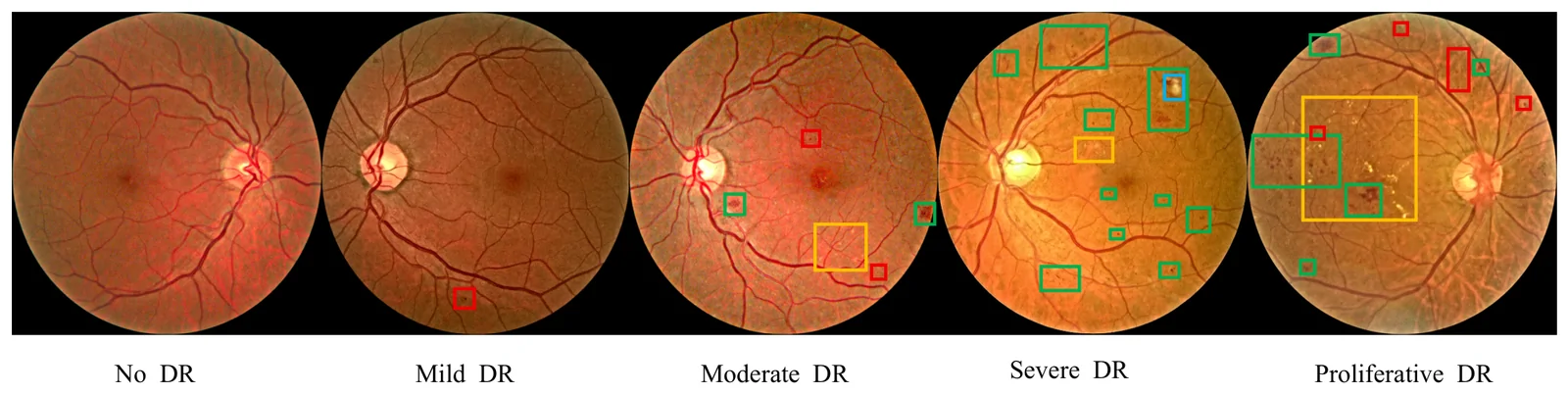
Representative examples of typical lesions in diabetic retinopathy fundus images. Colored bounding boxes indicate different DR-related lesion categories: red boxes denote microaneurysms (MA), green boxes denote hemorrhages (HE), yellow boxes denote hard exudates (EX), and blue boxes denote soft exudates (SE).

Diabetic retinopathy (DR) is among the most common and severe microvascular complications of diabetes and remains a leading cause of visual impairment and irreversible blindness in working-age populations worldwide [1]. As the global prevalence of diabetes continues to increase, epidemiological studies predict that the number of individuals affected by DR will reach approximately 160.5 million by 2045 [2]. This rapidly growing disease burden poses substantial challenges not only to public health systems and ophthalmic care resources but also to the capacity of primary-care services to conduct large-scale screening. In clinical practice, DR severity is commonly assessed according to the International Clinical Diabetic Retinopathy Severity Scale (ICDR) [3]. Based on the type, number, and spatial distribution of retinal lesions, DR is generally categorized into five ordered grades: no DR, mild DR, moderate DR, severe DR, and proliferative DR, as summarized in Table 1. These five grades reflect a continuous pathological process that begins with microaneurysm formation, progresses through hemorrhage, exudation, and ischemic changes, and may eventually lead to neovascularization and vitreous or preretinal hemorrhage [4]. In color fundus images, this progression is manifested by characteristic retinal lesions, including microaneurysms, dot-and-blot hemorrhages, hard exudates, soft exudates, and neovascularization [5]. Representative examples are shown in Figure 1. Despite the clinical importance of accurate DR grading, conventional diagnosis still relies primarily on the manual interpretation of fundus images by ophthalmologists. Because this process requires considerable medical expertise and resources, while also being affected by differences in physician experience, fatigue, and inter-observer variability, its efficiency and reliability may be limited in large-scale screening settings [6]. Consequently, automated DR grading has become an important research direction in medical image analysis and intelligent ophthalmic diagnosis.

In recent years, deep learning methods have achieved substantial progress in automated DR grading. Convolutional neural network (CNN)-based approaches [7,8,9,10], which are capable of extracting local textures and lesion-related features from fundus images, have demonstrated promising performance on multiple public datasets. However, because CNNs primarily rely on local convolutional operations, their ability to capture correlations among spatially separated lesions and to model global lesion distributions remains limited. In contrast, transformer-based methods [11,12,13,14] use self-attention mechanisms to model global contextual information, allowing them to capture latent dependencies among different retinal regions more effectively. However, transformers may still struggle to reliably identify small-scale or low-contrast lesions and to preserve fine-grained local texture information. Although CNNs and Transformers provide complementary advantages in local feature extraction and global dependency modeling, both types of methods remain predominantly driven by visual signals. As a result, they lack explicit guidance from clinical semantic priors related to lesion regions and disease severity. This limitation may restrict their ability to interpret complex lesion semantics. Consequently, it may reduce both their adaptability across datasets and the interpretability of their predictions in terms of underlying disease phenotypes.

More recently, vision–language models have provided a new perspective on automated DR grading. By aligning fundus images with clinical textual descriptions, these models can incorporate prior knowledge of lesion types, severity grades, and diagnostic semantics, thereby alleviating some of the limitations of purely visual approaches in clinical semantic understanding. As a representative contrastive vision–language pretraining model, CLIP [15] learns transferable cross-modal representations by aligning image features with textual descriptions and has achieved promising performance in various downstream vision tasks. However, because its pretraining objective is primarily designed to align an entire image with its corresponding text, CLIP lacks explicit mechanisms for modeling fine-grained semantic structures within an image. Therefore, although CLIP is effective in generalized semantic understanding and cross-modal association, its reliance on image-level alignment limits its ability to recognize subtle and localized abnormalities in medical images. Retina-specific vision–language pretraining further improves the applicability of such models to ophthalmic image analysis. In particular, RetiZero [16] learns rich ophthalmic vision–language semantic priors through large-scale pretraining on fundus images and clinical texts. It has demonstrated strong performance in fundus disease recognition, image retrieval, and cross-domain generalization. Although the semantic knowledge learned by RetiZero provides a valuable foundation for DR grading, its original training objective primarily focuses on global image–text alignment. Consequently, RetiZero does not explicitly model the fine-grained differences among retinal lesions, the ordinal relationships among the five DR severity grades, or the substantial phenotypic heterogeneity that may exist within the same grade.

To address the above challenges, based on the semantic prior information provided by RetiZero, we propose a text-guided lesion mining vision–language ordinal classification framework for DR grading, which jointly models lesion region focus, grade order and intra-class phenotypic differences. First, guided by the ICDR criteria and clinical descriptions of typical DR lesions, we construct lesion-related textual prompts and design a Text-Guided Cross-Layer Lesion Mining module (TG-CLM). By exploiting differences in semantic responses to normal-tissue and lesion-related textual prompts, TG-CLM generates soft lesion masks that highlight potentially abnormal retinal regions. Through further cross-modal interaction, TG-CLM guides multi-level visual patch features to focus on lesion regions that are particularly relevant to DR grading. Second, because DR severity follows an inherently ordered progression, we introduce a conditional ordinal regression branch that constructs an ordinal probability distribution consistent with the progression across DR severity grades. The resulting distribution is then used to perform Ordinal Distribution Alignment (ODA) on the vision–language classification branch, through which the predictions are encouraged to preserve the ordinal relationships among adjacent severity grades. Finally, to account for the substantial phenotypic variations that may occur within the same DR grade, we propose a Multi-Center Feature Constraint (MFC). Rather than representing each severity grade with a single feature center, MFC learns multiple phenotypic centers for each grade, allowing it to characterize the multimodal feature distributions induced by different lesion combinations at the same severity level. In this way, the adverse effect of intra-grade heterogeneity on the classification of borderline samples can be effectively mitigated.

The main contributions of this work are summarized as follows:

(1) We propose a text-guided lesion mining vision–language ordinal classification framework for DR grading, which jointly models lesion region focus, grade order and intra-class phenotypic differences.
(2) To enhance fine-grained lesion perception, we develop a text-guided cross-layer lesion mining module that exploits semantic response differences between normal-tissue and lesion-related textual prompts, thereby guiding multi-level visual patch features to focus on lesion regions relevant to DR grading.
(3) To explicitly model the ordered progression of DR severity, we introduce a conditional ordinal regression branch that constructs an ordinal probability distribution consistent with the relationships among DR severity grades. Based on this distribution, we further employ an ordinal distribution alignment strategy to constrain the outputs of the vision–language classification branch, thereby better preserving the ordinal structure of DR severity in its predictions.
(4) To alleviate intra-grade phenotypic heterogeneity, we introduce a multi-center feature constraint that learns multiple phenotypic centers for each severity grade, thereby improving the representation of diverse lesion combinations and enhancing the discrimination of borderline samples.

## 2. Related Work

### 2.1. Deep Learning-Based DR Grading Methods

Yang et al. [17] developed a two-stage deep convolutional network that first detected lesion regions and then integrated local and global information for DR grading, demonstrating the importance of lesion localization. Wu et al. [18] proposed the coarse-to-fine CF-DRNet, which first distinguished referable DR from non-DR cases and then performed fine-grained severity classification, thereby better reflecting the hierarchical nature of DR grading. To improve fine-grained lesion detection, subsequent studies incorporated attention mechanisms and multiscale feature fusion. Li et al. [19] introduced a lesion-attention pyramid network that extracted multiscale features from multi-resolution subnetworks and used lesion activation maps to guide high-resolution feature learning. Yue et al. [20] designed an attention-driven cascaded network to propagate shallow lesion-aware cues to high-level features, while Bhati et al. [21] employed bidirectional spatial and channel-parallel attention to emphasize lesion-related regions and informative channels.

To overcome the limitations of CNNs in modeling global context, Transformers have been introduced into the DR classification task. Sun et al. [22] proposed a lesion-aware Transformer for joint DR grading and weakly supervised lesion discovery, incorporating lesion-importance and lesion-diversity mechanisms to identify discriminative regions. Gu et al. [23] combined a vision Transformer with residual attention to focus on key lesions such as hemorrhages and exudates, whereas Zang et al. [24] introduced category-relationship attention to model intra-class features and global spatial relationships among lesions.

In recent years, several studies have sought to combine the strengths of CNNs and Transformers to enhance both local lesion feature extraction and global contextual modeling. Huang et al. [25] proposed DR-CTFN, which integrated ConvNeXt, Swin Transformer, and lightweight attention to improve feature representation and cross-dataset generalization. Yi et al. [26] proposed CTANet, in which a CNN branch enhanced local features, a ViT branch captured global structure, and an adaptive focus fusion module integrated both types of information.

Overall, these methods have advanced local lesion modeling, multiscale feature fusion, and global context learning. Nevertheless, they remain primarily driven by visual supervision and make limited use of clinical textual semantics related to lesion types and disease severity. Moreover, few methods jointly model the ordinal progression of DR grades and intra-class phenotypic heterogeneity.

### 2.2. Vision–Language Models in Ophthalmic Image Analysis

Inspired by CLIP [15], medical vision–language models have been increasingly adopted for cross-modal medical representation learning. Wang et al. [27] proposed MedCLIP, which extended contrastive vision–language learning to medical image analysis and alleviated the scarcity of paired medical image–text data by leveraging unpaired medical images and texts. Du et al. [28] proposed RET-CLIP, which was pretrained on retinal images and corresponding clinical diagnostic reports to learn retinal representations enriched with clinical diagnostic semantics. Shi et al. [29] proposed EyeCLIP for multimodal ophthalmic image analysis, incorporating self-supervised reconstruction, multimodal image contrastive learning, and image–text contrastive learning to learn shared multimodal representations, thereby improving few-shot learning and cross-domain generalization.

In retinal image analysis, Wang et al. proposed RetiZero [16], a vision–language model pretrained on large-scale fundus images and corresponding clinical texts to learn rich ophthalmic disease semantics. RetiZero has demonstrated strong performance in common and rare fundus disease recognition, image retrieval, few-shot classification, and cross-domain generalization. For DR grading, it provides valuable ophthalmic vision–language semantic priors that enhance the understanding of lesion-related clinical semantics.

However, most existing ophthalmic vision–language models primarily focus on global image–text semantic alignment and general fundus disease recognition, without explicitly modeling fine-grained lesion differences, ordinal grade relationships, or intra-class phenotypic heterogeneity in five-level DR grading. In particular, different DR grades often share similar lesion types, whereas the same grade may result from different lesion combinations. Therefore, relying solely on global image–text alignment is insufficient to fully capture the fine-grained correspondence between lesion-level semantic evidence and disease severity.

### 2.3. Ordinal Regression for Disease Severity Prediction

Cao et al. [30] proposed the Consistent Rank Logits (CORAL) framework, which transforms the ordinal learning objective into a set of binary classification subtasks. By imposing weight-sharing constraints on the output layer and introducing ordered bias terms, CORAL enforces rank monotonicity across binary subtasks and alleviates rank inconsistency in ordinal prediction. However, its ranking consistency mainly relies on output-layer weight sharing. When different disease stages correspond to complex and nonlinear pathological phenotypes in medical images, this shared-weight assumption may limit the representation of stage-specific visual patterns.

To improve the flexibility of ordinal modeling, Shi et al. [31] proposed CORN, a conditional probability-based ordinal regression method that maintains rank consistency without explicit constraints. CORN models conditional dependencies among severity levels by sequentially predicting whether a sample exceeds each severity threshold. This property makes it suitable for ordered classification tasks with progressive patterns, such as disease severity assessment.

In DR grading, Ma et al. [32] formulated DR grading as a joint ordinal regression and multi-class classification problem and proposed a KC loss to capture both class-level and ordinal supervision. Yu et al. [33] proposed AOR-DR, an autoregressive ordinal regression method that decomposes DR grading into a sequence of ordered prediction steps and conditions each step on the previous prediction and image features. In addition, AOR-DR employs a diffusion process to facilitate conditional probability modeling, enabling autoregressive prediction from continuous global image features without relearning contextual information from patch-level features. Yu et al. [34] further proposed a ranking-aware prompt strategy based on vision–language models, in which learnable prompts are designed between adjacent text–image pairs and ordinal information is exploited in different ranking directions to improve DR grading performance.

Overall, existing ordinal regression methods effectively utilize ordered relationships among disease grades and mitigate the limitation of conventional multi-class classification methods that ignore label order. However, most methods introduce ordinal constraints mainly at the label-space or prediction-output level, without fully integrating lesion-related textual semantics, cross-layer visual lesion evidence, and intra-grade multi-phenotype distributions.

## 3. Methods

### 3.1. Method Overview

**Figure 2:**
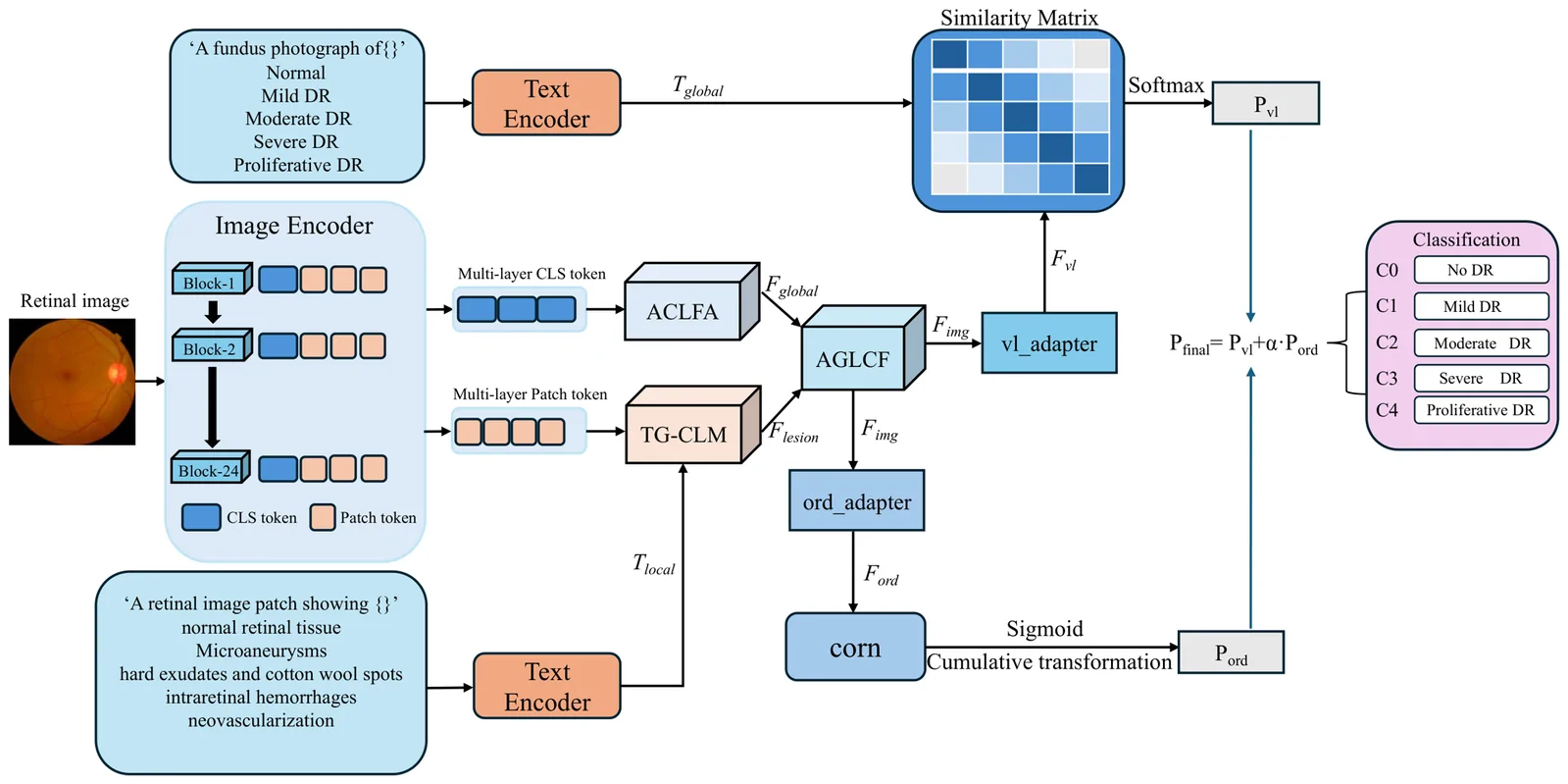
Overall architecture of the proposed model. $T_{global}$ denotes the global text embeddings generated from diabetic retinopathy severity-level prompts, while $T_{local}$ denotes the local text embeddings generated from retinal lesion-specific prompts.

As shown in Figure 2, this paper proposes a diabetic retinopathy (DR) grading framework based on text-guided cross-layer lesion mining and ordinal regression constraints. The framework adopts the pretrained ophthalmic vision–language model RetiZero as the backbone for vision–language feature extraction. Both the visual and text encoders are frozen during training. Inspired by CLIP-Adapter [35], we introduce two independent branch-specific adapters, ${\mathrm{Adapter}}_{\mathrm{vl}}$ and ${\mathrm{Adapter}}_{\mathrm{ord}}$, on the visual side for task-adaptive feature adjustment in the vision–language classification branch and the ordinal regression branch, respectively. Each adapter adopts a two-layer bottleneck architecture of 1024→256→1024, with a reduction ratio of 4. Both linear layers are bias-free and followed by ReLU activation. The two adapters have identical architectures but do not share parameters. This design adapts the fused retinal representations to the five-level DR grading task while preserving the pretrained vision–language semantic priors.

To incorporate clinical knowledge into visual feature learning, we construct two types of textual prompts: global grade prompts and local lesion prompts. The global prompt template is “A fundus photograph of {}”, where the placeholder is filled with one of five severity descriptions: “normal”, “mild diabetic retinopathy”, “moderate diabetic retinopathy”, “severe diabetic retinopathy”, and “proliferative diabetic retinopathy”.

The local prompt template is “A retinal image patch showing {}”, where the placeholder is filled with “normal retinal tissue”, “microaneurysms”, “hard exudates and cotton-wool spots”, “intraretinal hemorrhages”, or “neovascularization”. The normal-tissue prompt acts as a semantic reference, while the remaining four prompts provide lesion-related semantic probes for TG-CLM.

Given an input color fundus image $I\in R^{H\times W\times 3}$, the visual encoder first extracts multi-level visual representations, including global CLS tokens and local patch tokens from different layers. The CLS tokens are aggregated by the Adaptive Cross-Layer Feature Aggregation (ACLFA) module to obtain global semantic features, whereas the patch tokens are processed by the Text-Guided Cross-Layer Lesion Mining (TG-CLM) module to extract lesion-aware representations under textual guidance. The global and lesion-aware features are then fused through the Adaptive Global–Local Channel Fusion (AGLCF) module to generate the final visual representation.

The fused features are optimized through a dual-branch prediction structure. The vision–language classification branch performs semantic matching with global prompts, whereas the ordinal regression branch models the ordered relationships among DR severity levels. The two prediction distributions are further combined to produce the final grading result.

### 3.2. Text-Guided Cross-Layer Lesion Mining Module

**Figure 3:**
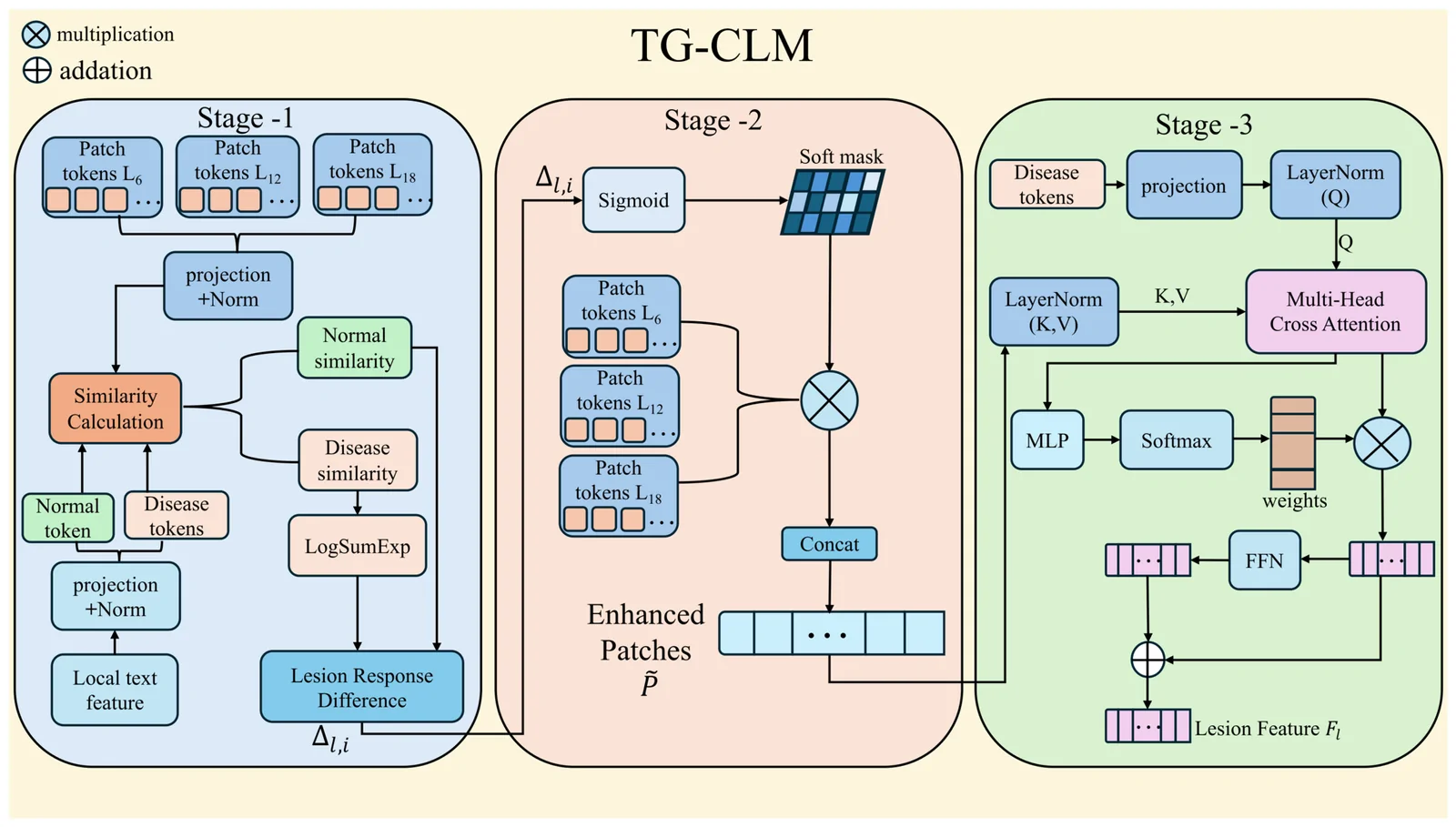
The detailed structure of TG-CLM.

To exploit clinical semantic priors for lesion-aware feature learning, we propose a Text-Guided Cross-Layer Lesion Mining module (TG-CLM), as shown in Figure 3. Unlike conventional attention mechanisms that select regions solely on the basis of visual features, TG-CLM uses normal-tissue and disease-related textual concepts as semantic anchors, thereby guiding the model to identify clinically relevant lesion regions. Specifically, TG-CLM first estimates the semantic discrepancy between disease-related and normal responses for each visual patch. It then generates lesion-aware soft masks to enhance pathological regions and finally aggregates disease-specific representations through text-guided cross-attention. The proposed module consists of three stages: multi-scale vision–text similarity modeling, lesion soft-mask generation and enhancement, and text-probe-guided lesion feature aggregation.

#### 3.2.1. Multi-Scale Vision–Text Similarity Modeling

Following the hierarchical design of the RetiZero visual encoder, patch tokens from the 6th, 12th, and 18th Transformer layers are selected for multi-level lesion mining. These layers capture visual representations at different semantic depths and constitute the three feature levels processed by TG-CLM. Specifically, each patch feature has a dimension of 1024.

Given the visual patch features extracted from the *l*-th selected layer, where l∈{6,12,18}, we denote them as $P_{l}\in R^{B\times N_{l}\times D_{v}}$, where *B*, $N_{l}$, and $D_{v}=1024$ represent the batch size, the number of patch tokens, and the visual feature dimension, respectively. The local textual features are denoted as $T\in R^{C\times D_{t}}$, where *C* is the number of textual concepts and $D_{t}$ is the text feature dimension. To project visual and textual features into a shared semantic space, feature transformation and $L_{2}$ normalization are first performed:

(1) $${\hat{P}}_{l}=\ell_{2}\left(P_{l}W_{v}^{l}\right),\;\hat{T}=\ell_{2}\left(TW_{t}\right),$$

where $W_{v}^{l}\in R^{D_{v}\times d}$ and $W_{t}\in R^{D_{t}\times d}$ are projection matrices, and $\ell_{2}\left(\cdot \right)$ denotes feature-wise $L_{2}$ normalization. The normalized textual embeddings are divided into a normal semantic token ${\hat{T}}^{n}$ and disease-related semantic tokens ${\left\{{\hat{T}}_{c}^{d}\right\}}_{c=1}^{C-1}$. For the *i*-th visual patch at layer *l*, the semantic similarity with normal and disease concepts is calculated as:

(2) $$S_{l,i}^{n}={\hat{P}}_{l,i}^{⊤}{\hat{T}}^{n},\;S_{l,i,c}^{d}={\hat{P}}_{l,i}^{⊤}{\hat{T}}_{c}^{d},$$

where ${\hat{P}}_{l,i}$ denotes the *i*-th patch feature in the shared semantic space. The dot product measures the semantic alignment between visual patches and textual concepts. Since multiple lesion types may simultaneously appear in a fundus image, a LogSumExp (LSE) aggregation strategy is adopted to integrate different disease-related semantic responses:

(3) $${\bar{S}}_{l,i}^{d}=\tau_{LSE}\log\left(\sum_{c=1}^{C-1}\exp\left(\frac{S_{l,i,c}^{d}}{\tau_{LSE}}\right)\right),$$

where $\tau_{LSE}>0$ controls the smoothness of semantic aggregation. The lesion relevance score of each patch is then calculated by the semantic discrepancy between disease and normal responses:

(4) $$\Delta_{l,i}={\bar{S}}_{l,i}^{d}-S_{l,i}^{n}.$$

A higher $\Delta_{l,i}$ indicates stronger alignment between the visual patch and disease-related concepts, whereas a lower value suggests normal retinal regions. By introducing normal-tissue semantics as a reference, TG-CLM reduces interference from normal retinal structures and highlights potential lesion regions.

#### 3.2.2. Lesion Soft-Mask Generation and Enhancement

Visual features from different layers contain diverse spatial resolutions and semantic levels. Therefore, layer-specific learnable parameters are introduced to adaptively calibrate lesion responses. The lesion soft mask at layer *l* is generated as:

(5) $$M_{l,i}=\sigma \left(\gamma_{l}\left(\Delta_{l,i}-b_{l}\right)\right),\;\gamma_{l}=\mathrm{Softplus}\left(\theta_{l}\right)+1,$$

where $\theta_{l}$ and $b_{l}$ are learnable parameters, σ(·) denotes the sigmoid function, and $\gamma_{l}$ controls the response sharpness. The generated soft mask is then applied to reweight patch features:

(6) $${\tilde{P}}_{l,i}=P_{l,i}⊙M_{l,i},$$

where ⊙ denotes element-wise multiplication, $M_{l,i}$ represents the lesion activation weight of the corresponding patch. This operation enhances lesion-related features while suppressing irrelevant background information. The enhanced patch features from multiple selected layers are concatenated along the token dimension to obtain a unified multi-scale lesion candidate representation:

(7) $$\tilde{P}=Concat\left({\tilde{P}}_{l_{1}},{\tilde{P}}_{l_{2}},\ldots ,{\tilde{P}}_{l_{m}}\right).$$

#### 3.2.3. Text-Probe-Guided Lesion Feature Aggregation

After obtaining lesion-aware candidate features, TG-CLM further employs disease-related textual embeddings as semantic probes to retrieve lesion representations associated with specific pathological concepts. The disease textual embeddings are projected into the visual feature space:

(8) $$Q={\hat{T}}^{d}W_{t\to v},$$

where $W_{t\to v}$ denotes the text-to-visual projection matrix, and *Q* represents disease semantic queries. A multi-head cross-attention mechanism is then applied, where disease queries attend to lesion candidate features:

(9) $$E=\mathrm{MHCA}(Q,\tilde{P},\tilde{P}),$$

where $E=[E_{1},E_{2},\ldots ,E_{C-1}]$ denotes the retrieved disease-specific lesion representations. Because different lesion types contribute differently to DR grading, an adaptive probe weighting strategy is introduced. Each disease probe is assigned an importance score and normalized through Softmax. The final lesion representation is obtained by weighted aggregation:

(10) $$a_{c}=f_{s}\left(E_{c}\right),\;\alpha_{c}=\frac{\exp(a_{c})}{\sum_{k=1}^{C-1}\exp\left(a_{k}\right)},\;F_{\mathrm{lesion}}=\sum_{c=1}^{C-1}\alpha_{c}E_{c},$$

where $f_{s}\left(\cdot \right)$ is a lightweight scoring network and $\alpha_{c}$ denotes the contribution of the *c*-th disease probe. This adaptive aggregation enables the model to dynamically emphasize disease-related semantics according to different retinal conditions.

Finally, a feed-forward network with residual connection is employed to refine the lesion representation:

(11) $$F_{lesion}^{out}=F_{lesion}+\mathrm{FFN}\left(F_{lesion}\right),$$

where the FFN consists of two linear layers with GELU activation and dropout. The resulting feature $F_{lesion}^{out}$ is used as the local lesion representation for subsequent global–local feature fusion.

In our implementation, the visual feature dimension is $D_{v}=1024$, the text feature dimension is $D_{t}=768$, and the shared vision–language projection dimension is d=512. The LogSumExp temperature is fixed at $\tau_{LSE}=0.1$. The layer-specific soft-mask parameters $\theta_{l}$ and $b_{l}$ are initialized to 2.0 and 0.1, respectively. text-guided lesion aggregation is implemented using an eight-head cross-attention layer with a dropout rate of 0.1. The subsequent FFN has dimensions 1024→2048→1024 and uses GELU activation and a dropout rate of 0.1.

### 3.3. Adaptive Global–Local Feature Fusion

Local lesion representations extracted by TG-CLM provide discriminative pathological cues for diabetic retinopathy (DR) grading. However, relying only on local features may neglect global retinal structures and contextual information. What is more, global CLS tokens capture high-level semantic representations of the entire fundus image but may contain redundant information and insufficient lesion-specific details. Therefore, we propose an Adaptive Global–Local Feature Fusion module to integrate complementary global semantic context and local lesion evidence.

**Figure 4:**
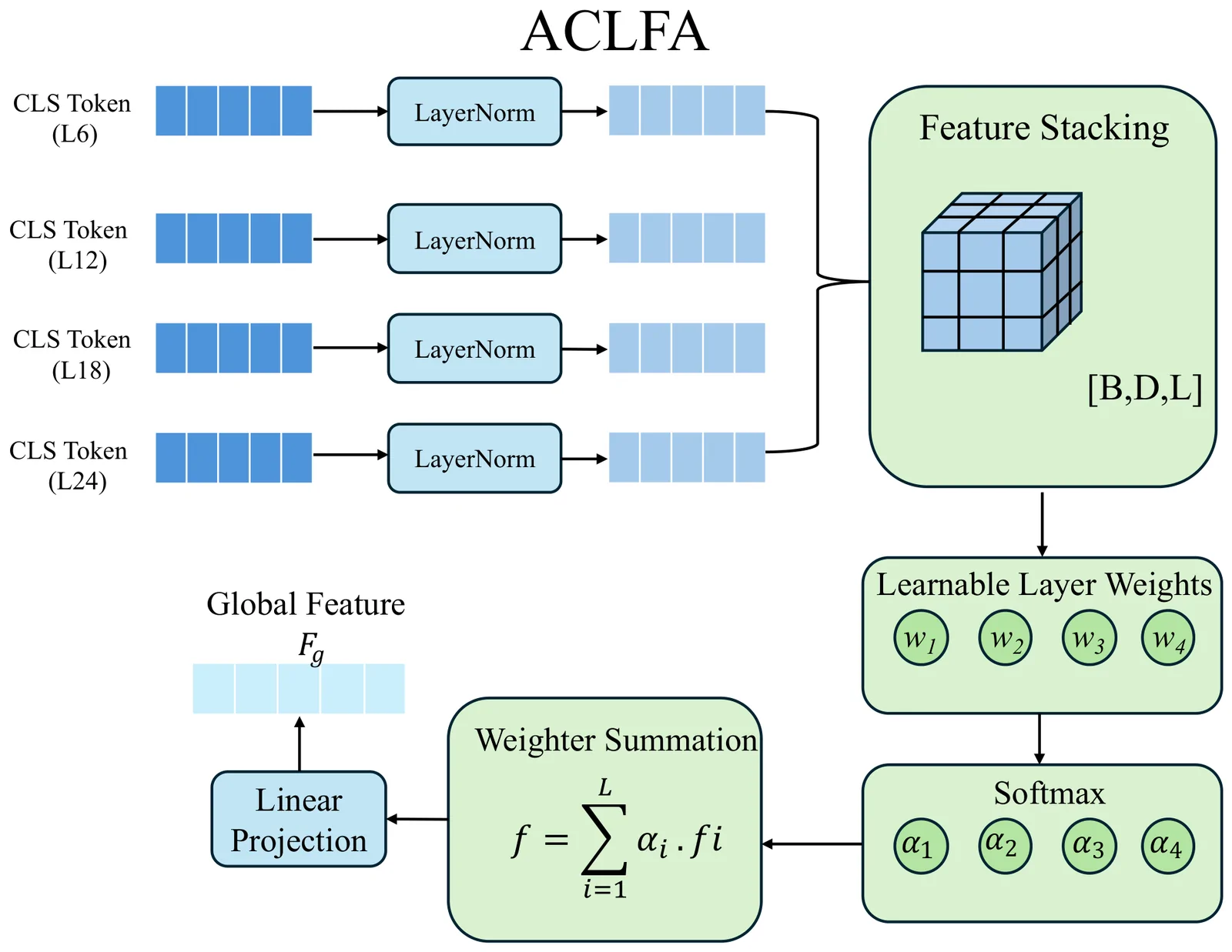
The structure of ACLFA.

First, an Adaptive Cross-Layer Feature Aggregation (ACLFA) module, as shown in Figure 4, is introduced to aggregate CLS tokens from multiple layers. Specifically, the global feature representation is obtained through learnable layer-wise weighting:

(12) $$f_{g}=\sum_{l\in L_{g}}\alpha_{l}\mathrm{LN}\left(g^{l}\right),\;\alpha_{l}=\frac{\exp(w_{l})}{\sum_{j\in L_{g}}\exp\left(w_{j}\right)},$$

where $L_{g}$ denotes the set of Transformer layers selected for global feature aggregation. In our implementation, we set $L_{g}=6,12,18,24$, indicating that the CLS tokens from the 6th, 12th, 18th, and 24th Transformer layers are aggregated. Here, $g^{l}$ represents the CLS token extracted from the *l*-th layer, and LN(·) denotes layer normalization. The learnable scalar $w_{l}$ determines the contribution of each layer, while $\alpha_{l}$ denotes the corresponding normalized importance weight. The aggregated global feature $f_{g}$ and the local lesion representation $f_{l}$ obtained from TG-CLM are first projected into the same feature space and normalized:

(13) $${\bar{f}}_{g}=\frac{f_{g}}{\left|\right|f_{g}{\left|\right|}_{2}},\;{\bar{f}}_{l}=\frac{f_{l}}{\left|\right|f_{l}{\left|\right|}_{2}}.$$

**Figure 5:**
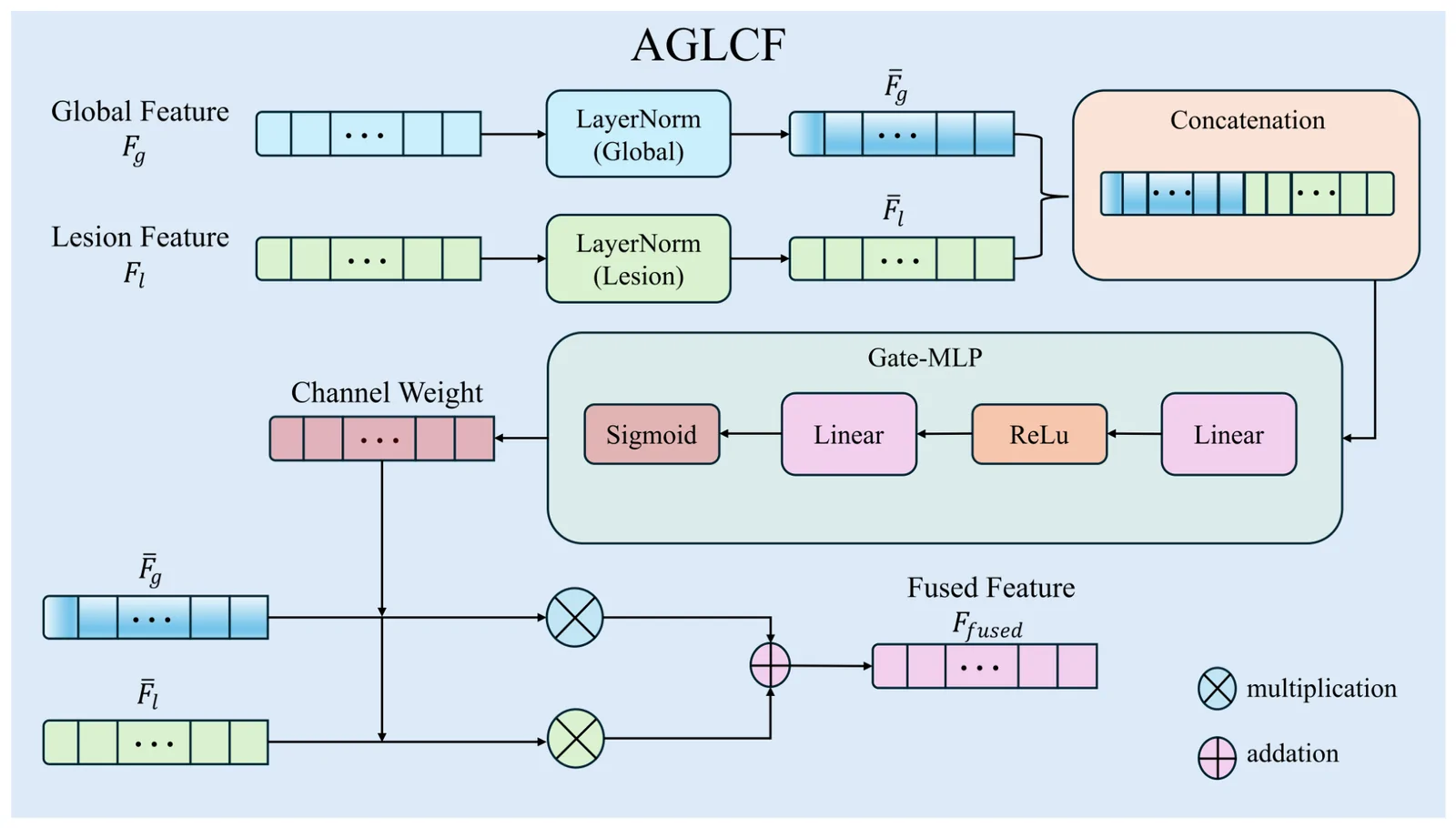
The structure of AGLCF.

To adaptively balance global context and lesion-specific information, an Adaptive Global–Local Channel Fusion (AGLCF) module is further proposed, as illustrated in Figure 5. The normalized global and local features are concatenated and fed into a lightweight gating network to generate channel-wise fusion weights:

(14) $$\omega =\sigma \left(\mathrm{MLP}\left([{\bar{f}}_{g};{\bar{f}}_{l}]\right)\right),$$

where [·;·] denotes feature concatenation and ω represents the channel-wise gating vector. The final fused representation is calculated as:

(15) $$f_{img}=\left(1-\omega \right)⊙{\bar{f}}_{g}+\omega ⊙{\bar{f}}_{l},$$

where ⊙ denotes element-wise multiplication. The adaptive gating mechanism enables the model to dynamically adjust the contribution of global semantic information and local lesion evidence according to the characteristics of each input image, producing a more discriminative representation for DR grading.

### 3.4. Ordinal Distribution Alignment

Diabetic retinopathy (DR) grading exhibits an intrinsic ordinal structure, where the five severity levels represent a progressive disease continuum rather than independent categories. Adjacent grades often share similar pathological characteristics, making fine-grained discrimination challenging. For example, mild and moderate DR may both present microaneurysms, while the distinction between moderate and severe DR is mainly determined by lesion quantity, distribution, and vascular abnormalities. Conventional cross-entropy optimization treats each grade independently and may ignore these inherent ordinal relationships, resulting in clinically inconsistent predictions among neighboring severity levels. To incorporate severity progression into the prediction process, we introduce a conditional ordinal regression branch alongside the vision–language classification branch and propose an Ordinal Distribution Alignment (ODA) strategy. The ordinal branch learns the ordered structure of DR severity, while ODA transfers ordinal knowledge to regularize the vision–language prediction. In the vision–language branch, the fused representation $f_{img}$ is first adapted through a lightweight adapter:

(16) $$f_{vl}=f_{img}+\lambda_{vl}\cdot {Adapter}_{vl}\left(f_{img}\right),\;\lambda_{vl}=\sigma \left(a_{vl}\right),$$

where $\lambda_{vl}$ is a learnable gating coefficient. The adapted feature and global grade-level textual embeddings are projected into a shared vision–language space:

(17) $$v=W_{img}f_{vl},\;e_{c}=W_{txt}t_{c}^{g},$$

where $t_{c}^{g}$ denotes the textual prompt corresponding to the *c*-th DR grade. The projected features are normalized before similarity calculation:

(18) $$\hat{v}=\frac{v}{{\left||v|\right|}_{2}},\;{\hat{e}}_{c}=\frac{e_{c}}{\left|\right|e_{c}{\left|\right|}_{2}}.$$

The vision–language logits are computed as:

(19) $$z_{c}^{vl}=\exp\left(s\right){\hat{v}}^{⊤}{\hat{e}}_{c},$$

where *s* is a learnable temperature parameter. The categorical probability distribution is obtained by:

(20) $$p_{c}^{vl}=\frac{\exp(z_{c}^{vl})}{\sum_{j=0}^{K-1}\exp\left(z_{j}^{vl}\right)}.$$

For ordinal modeling, another lightweight adapter is introduced:

(21) $$f_{ord}=f_{img}+\lambda_{ord}\cdot {Adapter}_{ord}\left(f_{img}\right),\;\lambda_{ord}=\sigma \left(a_{ord}\right).$$

For *K* ordered grades, the ordinal branch predicts K−1 threshold logits $r=[r_{0},r_{1},\ldots ,r_{K-2}]$. Each logit represents the conditional probability of exceeding a specific severity threshold:

(22) $$q_{k}=\sigma \left(r_{k}\right),\;k=0,\ldots ,K-2.$$

The ordinal probability distribution is reconstructed through conditional probability decomposition:

(23) $$\begin{array}{cc} p_{0}^{ord}\; & =1-q_{0},\\ p_{c}^{ord}\; & =\left(\prod_{k=0}^{c-1}q_{k}\right)\left(1-q_{c}\right),\;c=1,\ldots ,K-2,\\ p_{K-1}^{ord}\; & =\prod_{k=0}^{K-2}q_{k}. \end{array}$$

During training, the original class label is transformed into ordered binary supervision. For a sample with label $y_{i}$, the ordinal target $o_{i,k}^{*}$ indicates whether the sample exceeds the *k*-th severity threshold. A masking strategy is applied to include only valid ordinal comparisons:

(24) $$L_{ord}=\frac{\sum_{i=1}^{B}\sum_{k=0}^{K-2}m_{i,k}\left[-o_{i,k}^{*}\log q_{i,k}-\left(1-o_{i,k}^{*}\right)\log\left(1-q_{i,k}\right)\right]}{\sum_{i=1}^{B}\sum_{k=0}^{K-2}m_{i,k}},$$

where $m_{i,k}$ denotes the valid threshold mask. Although the ordinal branch captures disease progression, the vision–language branch provides stronger semantic interpretability through clinical text alignment. Therefore, ODA transfers ordinal knowledge from the ordinal branch to the vision–language branch by minimizing:

(25) $$L_{ODA}=\frac{1}{B}\sum_{i=1}^{B}D_{KL}\left(\mathrm{sg}\left(p_{i}^{ord}\right)\left|\right|p_{i}^{vl}\right),$$

where sg(·) denotes the stop-gradient operation. This asymmetric constraint enables the ordinal branch to serve as a teacher distribution and provide stable severity-aware supervision, while gradients do not flow back to the ordinal branch and only the vision–language prediction is regularized by this loss term.

### 3.5. Multi-Center Feature Constraint

DR grading suffers not only from inter-class similarity but also from substantial intra-class phenotypic variation. Samples within the same severity level may exhibit different combinations, distributions, and severities of retinal lesions. Therefore, forcing all samples of one grade toward a single feature prototype may suppress meaningful intra-class variations and reduce discrimination of borderline cases. To address this limitation, we propose a Multi-Center Feature Constraint (MFC), which represents each DR grade using multiple feature centers to model the multimodal phenotypic distribution caused by different lesion patterns. For the *c*-th class, a set of learnable feature centers is defined as: $C_{c}=\left\{\mu_{c,1},\mu_{c,2},\ldots ,\mu_{c,K_{c}}\right\}$, where $\mu_{c,k}\in R^{d}$ denotes the *k*-th phenotypic center of class *c*, and $K_{c}$ represents the number of centers assigned to that class. Given a feature representation $f_{i}\in R^{d}$ with ground-truth label $y_{i}$, MFC assigns the sample to the closest intra-class center rather than constraining it toward a single class prototype:

(26) $$L_{MFC}=\frac{1}{B}\sum_{i=1}^{B}\min_{1\leq k\leq K_{y_{i}}}\frac{1}{2}{∥f_{i}-\mu_{y_{i},k}∥}_{2}^{2},$$

where *B* is the batch size, $\mu_{y_{i},k}$ is the *k*-th phenotypic center of class $y_{i}$, and $K_{y_{i}}$ is the number of centers assigned to that class. This formulation allows multiple feature clusters to emerge within the same DR grade, thereby better representing diverse lesion phenotypes. During center updating, each sample is first assigned to its nearest center:

(27) $$k_{i}^{*}=\arg\min_{1\leq k\leq K_{y_{i}}}{∥f_{i}-\mu_{y_{i},k}∥}_{2}^{2}.$$

For each center, the assigned sample set is defined as: $B_{c,k}=\left\{\;i|y_{i}=c,\;k_{i}^{*}=k\;\right\}$. The centers are updated using the assigned samples:

(28) $$\mu_{c,k}\leftarrow \mu_{c,k}+\rho \frac{\sum_{i\in B_{c,k}}\left(f_{i}-\mu_{c,k}\right)}{\epsilon +|B_{c,k}|},$$

where ρ is the center update rate and ϵ is a small constant for numerical stability. If no samples are assigned to a center in the current batch, the corresponding center remains unchanged. By maintaining multiple centers for each severity level, MFC captures intra-grade phenotypic diversity and improves robustness against ambiguous samples.

### 3.6. Training and Inference Strategies

During training, the vision–language classification branch is optimized using cross-entropy loss:

(29) $$L_{cls}=-\frac{1}{B}\sum_{i=1}^{B}\log p_{i,y_{i}}^{vl}.$$

The overall training objective combines classification, ordinal regression, ordinal distribution alignment, and multi-center feature constraint:

(30) $$L=L_{cls}+L_{ord}+L_{ODA}+\gamma_{mfc}L_{MFC},$$

where $\gamma_{mfc}$ denotes the loss weight for the multi-center feature constraint term, whose specific value is provided in the experimental setup. During inference, the prediction score is computed by linearly combining the two distributions:

(31) $$p=p^{vl}+\alpha p^{ord},\;0\leq \alpha \leq 1,$$

where α is a fusion weight that balances the contribution of the ordinal branch. The final DR grade is determined by selecting the class with the highest fused score.

Through this training and inference strategy, the proposed framework jointly exploits clinical semantic knowledge from RetiZero, lesion-aware representations, ordinal severity relationships, and intra-grade phenotypic distributions, enabling more reliable discrimination of complex and borderline DR cases.

## 4. Experiments

### 4.1. Datasets and Data Preprocessing

To comprehensively evaluate the proposed method, we designed two experimental settings. First, we conducted a single-dataset evaluation on APTOS [36] to assess the intra-domain grading performance. Second, we further evaluated the cross-distribution robustness of the model by conducting domain generalization experiments on six public DR datasets, including APTOS, IDRiD [37], and others. The data distributions of these datasets are shown in Table 2, and detailed descriptions of each dataset are provided below:

**APTOS [36]:** These dataset was collected by Aravind Eye Hospital in India for the blindness detection competition at the 4th Asia-Pacific Telemedicine in Ophthalmology Society (APTOS) Symposium in 2019. It contains 3662 fundus images. For the single-dataset evaluation, we used a fixed random split generated with a fixed random seed, resulting in 2930 training images, 366 validation images, and 366 testing images. The exact class-wise composition of each subset is reported in Table 3. The same fixed partition was retained for all subsequent experiments to ensure consistent and fair comparisons.
**IDRID [37]:** These dataset is part of the “Diabetic Retinopathy: Segmentation and Grading Challenge” workshop at ISBI 2018. It includes lesion segmentation, disease grading, and optic disc/macula detection tasks. We used 516 images from Task 2.
**DEEPDR [38]:** These dataset was used for ISBI-2020 Challenge 5 (Grading and Diagnosis of Diabetic Retinopathy). This challenge contains regular fundus images and ultra-widefield images for different tasks. Following the adopted benchmark protocol, we only use 1980 images of the regular fundus images as the DeepDR dataset.
**MESSIDOR [39]:** It represents a method for evaluating segmentation and indexing techniques in the field of retinal ophthalmology. We use the Messidor-2 dataset. Compared to Messidor-1, Messidor-2 contains all images from the former as well as hundreds of additional images. In total, 1748 images are collected. To maintain consistency with the standardized domain-generalization protocol in GDRNet, we adopted the same 1744 images subset utilized in that benchmark. This guarantees identical target-domain data composition as employed for the reported baseline comparisons in GDRNet.
**FGADR [40]:** These dataset was collected by the Inception Institute of Artificial Intelligence (IIAI). It contains image-level grade labels and pixel-level lesion annotations.
**RLDR [41]:** These dataset is a subset of Eyepacs and contains 1593 images.

Since the six fundus image datasets used in the experiments were acquired using different devices, there are certain differences in lighting conditions, imaging quality, and resolution. To minimize the interference of these non-pathological factors on model training and prediction, we performed uniform preprocessing on the raw fundus images before feeding them into the network. We referenced the preprocessing code from EYEQ [42] to perform preprocessing operations, which involved removing black borders from the fundus images and cropping them to a size of 512×512. During the training phase, to further improve the model’s adaptability to different imaging conditions, we performed online data augmentation on the preprocessed fundus images. First, the images are resized to 256×256, and color perturbation is applied to randomly adjust brightness, contrast, saturation, and hue, thereby simulating color variations under different devices and lighting conditions. Next, drawing on GDRNet [43], we introduce fundus-image-specific enhancement operations, including sharpness variations, halos, local holes, speckle artifacts, and blurring, to simulate imaging degradations encountered in real-world clinical images. Finally, the images are randomly cropped to 224×224, undergo random horizontal and vertical flips, and are then normalized before being fed into the network.

### 4.2. Experimental Setup

All experiments were implemented in PyTorch 1.13.1 and conducted on an NVIDIA GeForce RTX 4090 GPU (NVIDIA Corporation, Santa Clara, CA, USA). The model was trained for 100 epochs with a batch size of 64. We adopted the AdamW optimizer with an initial learning rate of $1\times 10^{-4}$ and a weight decay of $1\times 10^{-5}$. During training, the RetiZero visual and text encoders were frozen. The model was jointly optimized with four loss functions: vision–language classification loss, conditional ordinal regression loss, ordinal distribution alignment (ODA) loss, and multi-center feature constraint (MFC) loss, with respective weights of 1.0, 1.0, 1.0, and 0.005. For the MFC module, the per-grade center configuration was set to [1,2,3,3,4] with a center update rate of 0.5. During inference, the final probability distribution was obtained by fusing the ordinal probability distribution with the vision–language probability distribution, where the weighting factor α was set to 0.7. The validation set was used exclusively for hyperparameter tuning and model selection, while the fixed APTOS 2019 test set (366 images) was reserved for final evaluation. To account for stochastic training variability, all controlled experiments on the APTOS 2019 dataset were repeated using the same set of five random seeds. Model performance was measured by accuracy, Macro-F1, macro-averaged one-vs-one AUC (Macro-OvO AUC), and class-wise one-vs-rest AUC. We further adopted quadratic weighted kappa (QWK), mean absolute grade error (MAE), within-one-grade accuracy (W1A), and cross-level error frequencies to evaluate ordinal consistency.

### 4.3. Comparison with Other Methods

**Figure 6:**
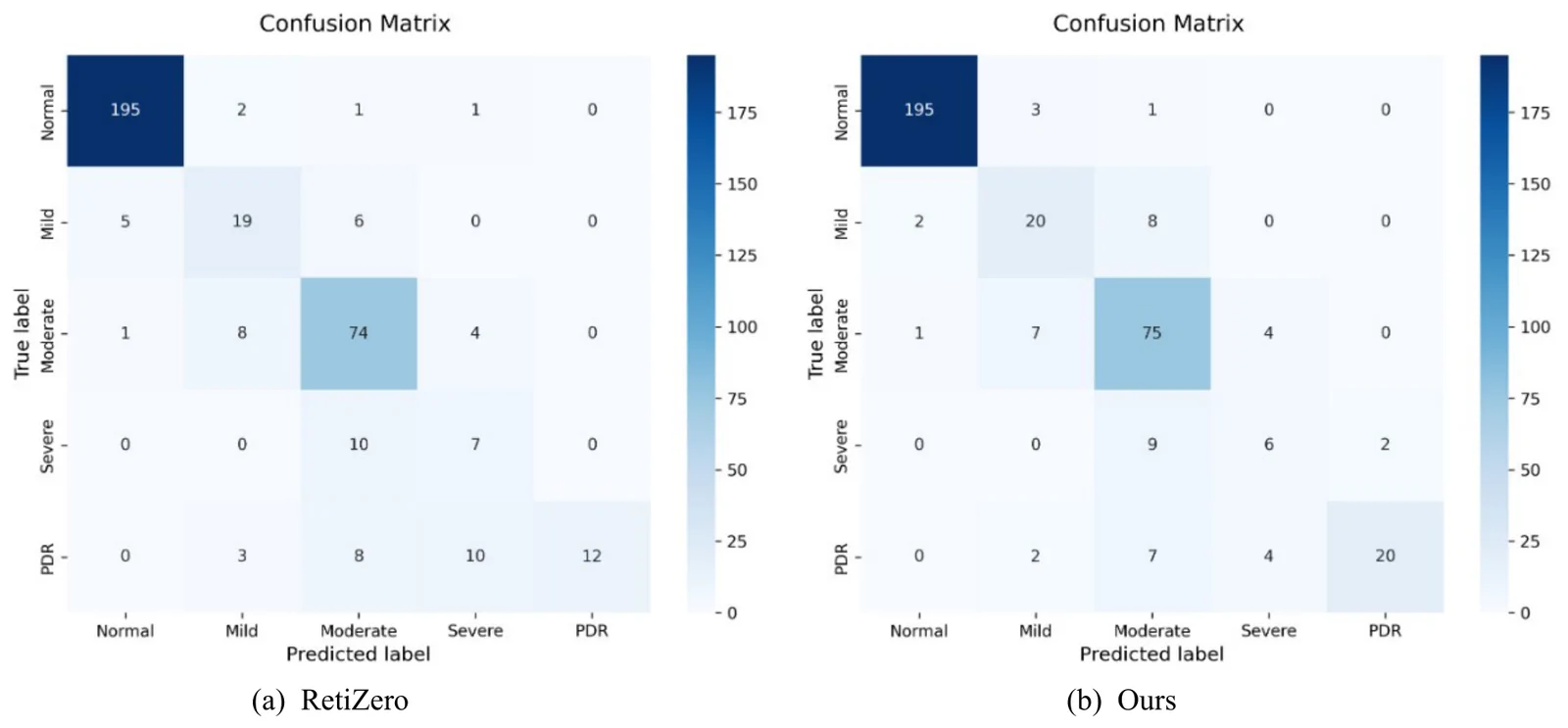
Confusion matrices of different methods evaluated on the independent APTOS 2019 test set (n=366).

**Figure 7:**
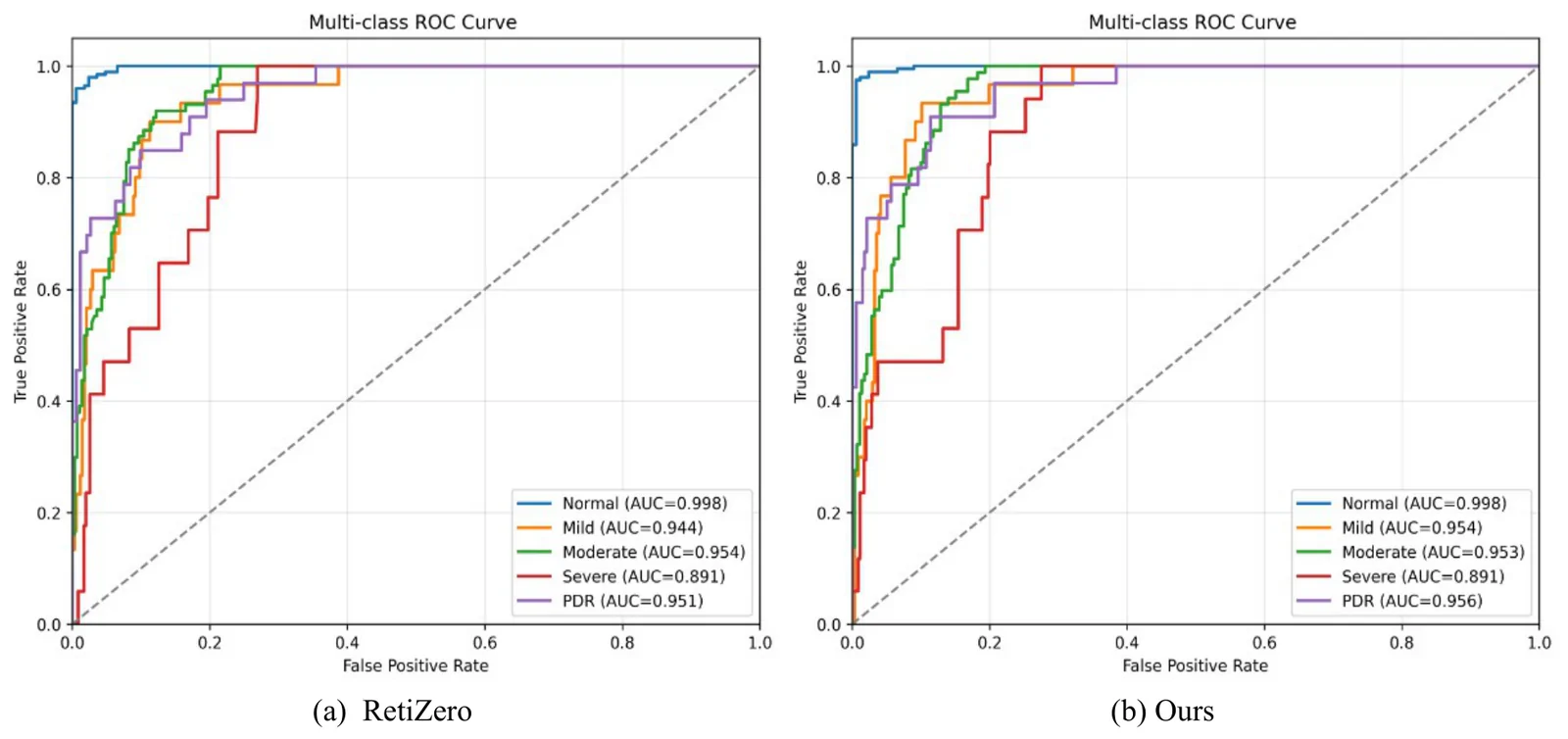
Class-wise one-vs-rest ROC curves of different methods on the APTOS 2019 dataset.

**Figure 8:**
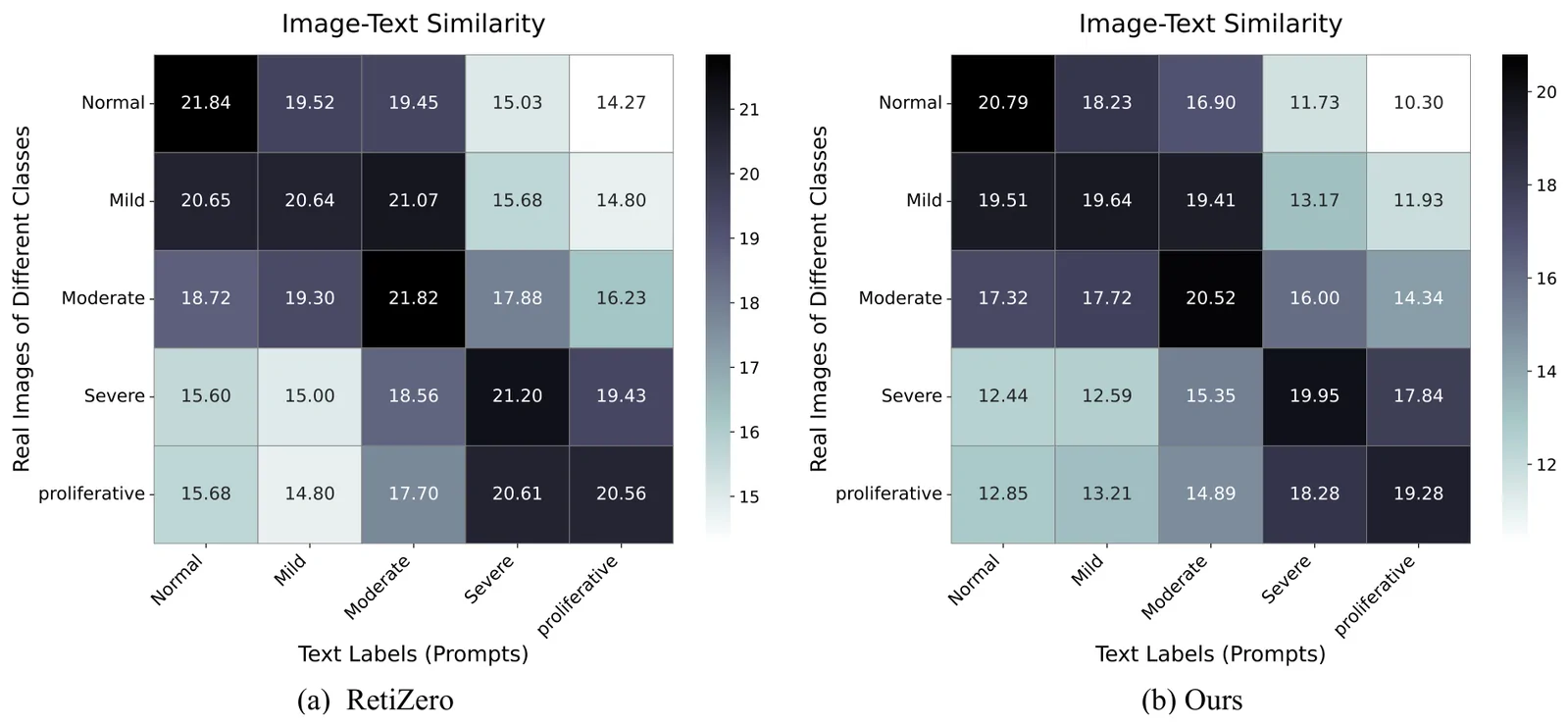
The image–text similarity matrix is obtained by the inner product between the image feature and the text feature: $X\cdot T^{⊤}$. This matrix provides an intuitive representation of the ranking relationships among different image–text pairs. The x-axis represents five different text labels, while the y-axis represents real images from different classes.

To evaluate the effectiveness of the proposed framework, we compare it with representative diabetic retinopathy grading methods on the APTOS 2019 dataset, including CNN-based, attention-based, Transformer-based, ordinal-regression, and vision–language approaches. The quantitative results are summarized in Table 4. For RetiZero, CLIP-DR, and AOR-DR, we report the results obtained under the same experimental setting as the proposed method, including the same fixed train/validation/test split, image preprocessing and augmentation pipeline, model-selection procedure, and evaluation metrics. The results of the remaining methods are taken from the corresponding published studies and are included as reference results because their experimental protocols may differ from ours. In addition, the confusion matrices, class-wise one-vs-rest ROC curves, and image–text similarity matrices of RetiZero and the proposed method are shown in Figure 6, Figure 7, and Figure 8, respectively.

As shown in Table 4, under the unified experimental setting, the proposed method achieves an average ACC of 86.3±1.2%, an AUC of 90.6±0.5%, and a Macro-F1 score of 70.9±2.0% over five runs. In comparison, RetiZero obtains 83.9±0.9%, 89.9±0.4%, and 65.4±1.9%, respectively. CLIP-DR achieves 82.6±1.6% ACC, 89.5±0.7% AUC, and 64.2±2.2% Macro-F1, whereas AOR-DR achieves 84.5±0.8%, 90.2±0.3%, and 68.1±1.2%, respectively. These results show that the proposed method maintains higher average performance across the five random-seed runs.

Compared with RetiZero, the proposed method improves ACC from 83.9% to 86.3%, AUC from 89.9% to 90.6%, and Macro-F1 from 65.4% to 70.9%, corresponding to improvements of 2.4, 0.7, and 5.5 percentage points, respectively. Compared with CLIP-DR, the proposed method improves ACC, AUC, and Macro-F1 by 3.7, 1.1, and 6.7 percentage points, respectively. Compared with AOR-DR, the corresponding improvements are 1.8, 0.4, and 2.8 percentage points. These results indicate that the proposed framework maintains consistent performance advantages over the closely related vision–language and ordinal-regression baselines when evaluated under the same data split and experimental protocol.

To quantify performance variability across stochastic training runs and further assess the statistical significance of the observed improvements, we conducted five repeated training and evaluation runs with different random seeds for the proposed method, RetiZero, CLIP-DR, and AOR-DR under the unified experimental protocol. The training/validation/test partition, preprocessing and augmentation pipeline, model-selection procedure, and evaluation metrics were kept identical across all runs. The same set of random seeds was used for corresponding runs of all compared methods, yielding five seed-matched paired observations for each model comparison and evaluation metric. For statistical comparisons, the performance difference between the proposed method and each baseline was calculated for each matched seed, and two-sided paired *t*-tests were subsequently performed. The significance level was set to α=0.05. Table 5 reports the mean ± standard deviation of these paired performance differences together with the corresponding *p*-values, with statistically significant differences ((*p* < 0.05)) indicated by an asterisk.

As shown in Table 5, the proposed method yields positive mean performance differences over all three controlled baselines across ACC, AUC, and Macro-F1. For example, compared with RetiZero, the proposed method improves ACC, AUC, and Macro-F1 by (2.40 ± 1.39), (0.70 ± 0.53), and (5.50 ± 2.59) percentage points across the five seed-matched runs, respectively. All paired comparisons yield (*p* < 0.05), indicating statistically significant differences under the unified experimental protocol.

For a more intuitive understanding of the classification results, the confusion matrices in Figure 6 provide a more detailed view of the classification behavior. For normal fundus images, both RetiZero and our method correctly classify 195 samples, indicating that the proposed modules preserve the strong normal-class recognition ability of the baseline. For mild DR, the number of samples misclassified as normal decreases from 5 to 2, suggesting that the proposed method reduces underestimation of early pathological cases. The most obvious improvement is observed for proliferative DR, where the number of correctly classified samples increases from 12 to 20. However, the improvement is not uniform across all advanced grades. For Severe DR, the number of correctly classified cases decreases slightly from 7 for RetiZero to 6 for the proposed method, with two Severe cases being overestimated as PDR. Meanwhile, long-distance misclassifications are reduced, and errors are mainly concentrated around adjacent grades. This indicates that the proposed framework is more sensitive to advanced pathological evidence, such as neovascularization and severe hemorrhagic changes.

To further assess the effectiveness of ordinal modeling, we evaluate ordinal prediction performance using quadratic weighted kappa (QWK), mean absolute grade error (MAE), within-one-grade accuracy (W1A), and long-distance error rates. In addition to RetiZero, we include AOR-DR, a dedicated ordinal-regression baseline evaluated under the same controlled protocol, to provide a more direct comparison of ordinal modeling capability.

As shown in Table 6, the proposed method increases QWK from 0.895 to 0.923 and reduces MAE from 0.210 to 0.172 compared with RetiZero. The reduction of 0.038 grade steps corresponds to an 18.2% relative decrease in the average absolute grade error, indicating that the predictions are, on average, closer to the ground-truth severity grades. W1A increases from 96.17% to 96.99%; equivalently, the number of predictions with an absolute grade error of at least two decreases from 14 of 366 cases (3.83%) to 11 of 366 cases (3.01%). For the more severe subset of errors spanning at least three grade steps, the number decreases from 4 cases (1.09%) to 2 cases (0.55%). Importantly, the proposed method also consistently outperforms the dedicated ordinal-regression baseline AOR-DR, increasing QWK from 0.905 to 0.923 and W1A from 96.72% to 96.99%, while reducing MAE from 0.197 to 0.172. The long-distance error rates are further reduced from 3.28% to 3.01% for errors of at least two grades and from 0.82% to 0.55% for errors of at least three grades. These observed results indicate that the complete framework achieves stronger agreement with the ordered DR grades and reduces large grade deviations on the current test set.

Nevertheless, misclassifications still mainly occur between adjacent grades, such as mild and moderate DR or moderate and severe DR. This is clinically reasonable because adjacent DR stages often share similar lesion types, and their differences may depend on lesion number, spatial distribution, and severity. In particular, severe DR remains challenging, suggesting that more precise modeling of lesion quantity and spatial distribution may further improve the discrimination of borderline cases.

Figure 7 shows the multi-class ROC curves of RetiZero and the proposed method. The proposed method achieves AUC values of 0.998, 0.954, 0.953, 0.891, and 0.956 for normal, mild DR, moderate DR, severe DR, and proliferative DR, respectively. Compared with RetiZero, the AUC values for mild DR and proliferative DR increase from 0.944 to 0.954 and from 0.951 to 0.956, respectively. These results indicate improved discrimination for both early-stage and advanced-stage DR, while maintaining strong separability for normal fundus images.

To further analyze the vision–language semantic alignment ability, we visualize the image–text similarity matrices of RetiZero and the proposed method in Figure 8. The vertical axis denotes fundus images from different DR classes, and the horizontal axis denotes text prompts of different severity levels. A discriminative vision–language grading model is expected to produce higher responses along the diagonal, where image classes and text prompts are semantically matched.

As shown in Figure 8a, RetiZero captures general image–text semantic correspondence, but its similarity distribution is not fully consistent with the ordinal structure of DR grading. For example, mild DR images show close responses to normal, mild, and moderate prompts, while proliferative DR images respond slightly more strongly to the severe DR prompt than to the proliferative DR prompt. This suggests that global image–text alignment alone may be insufficient to distinguish adjacent or visually similar severity levels.

In contrast, Figure 8b shows that the proposed method produces a more diagonal-consistent similarity pattern. For most DR grades, the highest similarity scores appear on the corresponding text prompts. In particular, the similarity score between proliferative DR images and the proliferative DR prompt becomes the highest in its row, which is consistent with the improved PDR classification shown in Figure 6. In addition, the responses of severe and proliferative DR images to normal and mild prompts are further suppressed, indicating that the proposed method reduces semantically unreasonable matching between distant severity levels.

Overall, the results on APTOS 2019 demonstrate that the proposed method achieves improved and more balanced DR grading performance under the controlled experimental setting. The quantitative comparison shows clear improvements in ACC and Macro-F1 over the controlled baselines, while the statistical analysis provides additional evidence regarding the reliability of these improvements across different random initializations. The confusion matrices indicate reduced underestimation of early-stage DR and improved recognition of PDR, the ordinal metrics show stronger agreement with the ordered severity structure, and the image–text similarity matrices suggest better alignment between fundus images and ordinal severity prompts. Together, these findings support the effectiveness of jointly modeling lesion-aware visual evidence, disease severity order, and intra-class phenotypic variation for five-level DR grading.

### 4.4. Domain Generalization Evaluation

To evaluate cross-dataset robustness, we conducted domain-generalization experiments on six public DR datasets, including APTOS, DeepDR, FGADR, IDRiD, Messidor, and RLDR. We adopted the standardized leave-one-domain-out evaluation protocol from GDRNet [43]. In each experiment, one dataset was treated as a completely unseen target domain, while the remaining five datasets were used as source domains for model training. No images or labels from the target domain were used for training, validation, hyperparameter selection, or model selection. The baseline results reported in Table 7 were taken from the published GDRNet benchmark rather than reproduced in the present study.

Following this benchmark protocol, all compared methods were evaluated with identical leave-one-domain-out source–target partitions as well as the standardized preprocessing, model-selection, and evaluation settings specified by GDRNet. The overall cross-dataset generalization results are summarized in Table 7.

As shown in Table 7, under the GDRNet benchmark protocol, the proposed method achieves the highest reported average ACC of 58.6%, which is 4.0 percentage points higher than the reported average ACC of PAF (54.6%). On APTOS, the proposed method obtains ACC, F1, and AUC values of 75.3%, 50.7%, and 86.3%, respectively. Compared with CLIP-DR, the F1 and AUC are higher by 4.4 and 3.0 percentage points, respectively. On IDRiD, the proposed method also achieves strong performance, with an ACC of 64.5%, an F1 score of 49.2%, and an AUC of 86.5%. These results suggest that the proposed framework has the potential to improve prediction performance when evaluated on previously unseen datasets.

However, the proposed method does not achieve the highest average F1 or average AUC across the six target domains. Therefore, the domain-generalization results should be interpreted as an improvement in average classification accuracy under the adopted benchmark rather than as comprehensive superiority over all compared methods.

**Figure 9:**
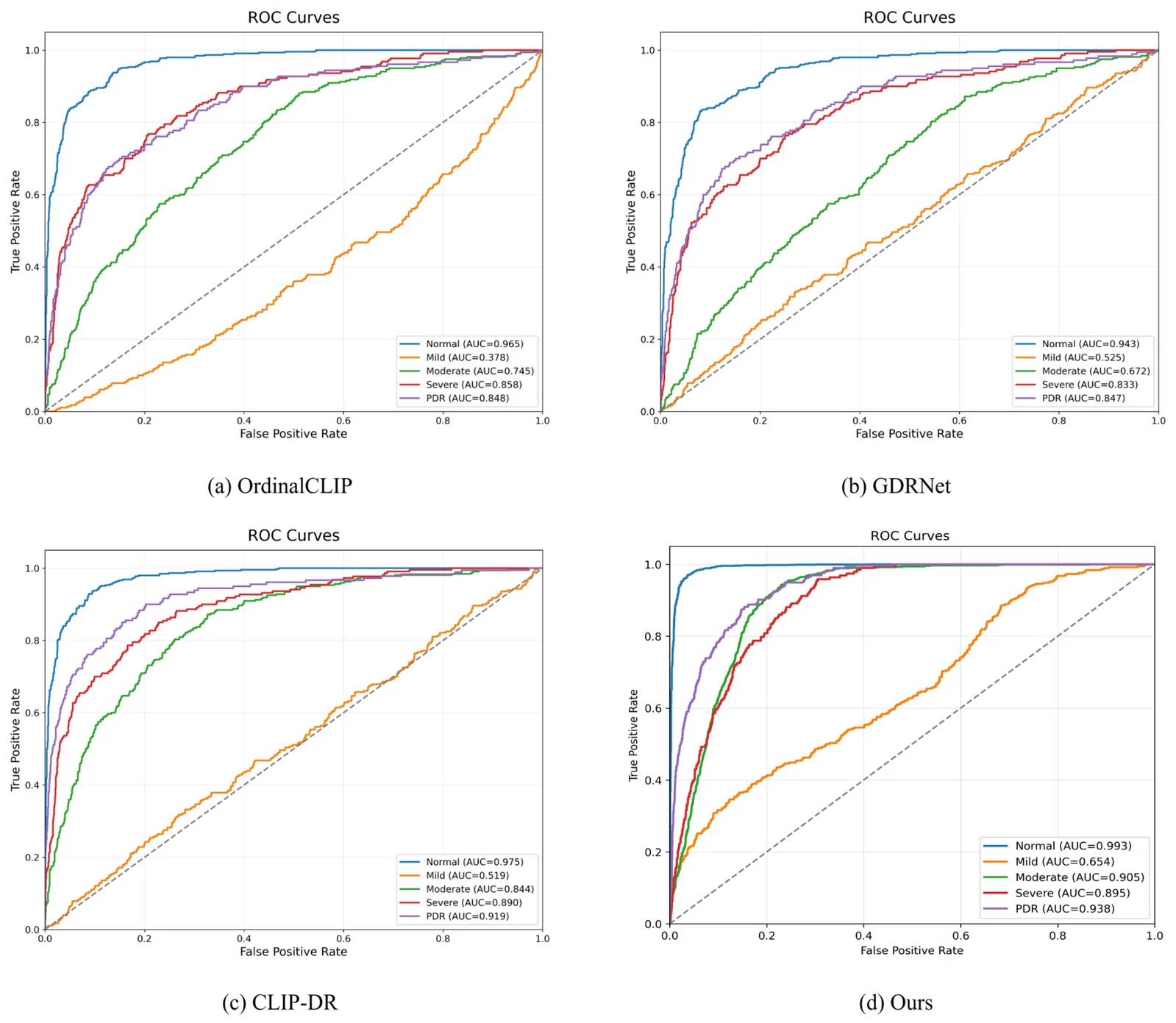
ROC curves of different methods on the APTOS target domain under the DG test.

Figure 9 further shows the class-wise one-vs-rest ROC curves when APTOS is used as the unseen target domain. Compared with OrdinalCLIP, GDRNet, and CLIP-DR, the proposed method shows better separability for most DR grades, particularly Normal, Moderate, Severe, and Proliferative DR. The Mild class remains challenging, reflecting the difficulty of identifying subtle early-stage pathological changes under domain shift.

The proposed method also exhibits limitations on several target domains. On FGADR, its ACC, F1, and AUC remain lower than those of the strongest competing methods. On Messidor, although the proposed method achieves the highest ACC of 65.1%, its F1 and AUC are lower than those of GDRNet and CLIP-DR. On RLDR, the method obtains a competitive AUC of 81.6%, whereas its ACC and F1 remain relatively low. These results indicate that large differences in imaging style, class distribution, image quality, and adjacent-grade appearance remain important challenges for generalizable DR grading.

### 4.5. Ablation Study

#### 4.5.1. Analysis of the Contribution of Each Module

To comprehensively evaluate the contributions of the proposed components, we conduct both an incremental ablation study and a dependency-aware controlled ablation study on the APTOS 2019 dataset. The RetiZero backbone equipped with a standard cross-entropy classification head is adopted as the baseline model. The text-guided cross-layer Lesion Mining module (TG-CLM), Conditional Ordinal Regression branch (CORN), Ordinal Distribution Alignment (ODA), and Multi-Center Feature Constraint (MFC) are first progressively incorporated according to the computational dependencies and construction sequence of the proposed framework. All reported values are averages over five runs using the same set of random seeds, and the quantitative results are summarized in Table 8.

As shown in Table 8, the baseline model achieves an ACC of 83.9%, an AUC of 89.9%, and a Macro-F1 of 65.4%. After TG-CLM is incorporated, these metrics increase to 84.5%, 90.3%, and 68.9%, respectively. In particular, Macro-F1 increases by 3.5 percentage points. This result suggests that lesion-related textual guidance provides useful complementary information for DR grading and is particularly helpful for improving balanced classification performance across the five severity grades.

Further incorporating the CORN branch increases ACC, AUC, and Macro-F1 to 85.7%, 90.5%, and 69.2%, respectively. Compared with Variant A, the corresponding improvements are 1.2, 0.2, and 0.3 percentage points. Unlike the conventional cross-entropy objective, CORN explicitly models the ordered relationships among DR severity grades. The observed improvements therefore suggest that ordinal supervision provides complementary severity information when combined with lesion-aware feature learning.

When ODA is subsequently introduced; however, the changes in the reported metrics are not uniformly positive. ACC increases slightly from 85.7% to 85.9%, whereas AUC decreases from 90.5% to 90.3% and Macro-F1 decreases from 69.2% to 68.5%. Therefore, the incremental results do not support a claim that ODA independently and consistently improves all evaluation metrics. Instead, its effect at this intermediate stage should be interpreted as a metric-dependent trade-off, suggesting that the contribution of ODA may depend on the surrounding model configuration.

After MFC is incorporated, the complete model achieves the best overall performance among the incremental configurations, with an ACC of 86.3%, an AUC of 90.6%, and a Macro-F1 of 70.9%. Compared with Variant C, MFC improves ACC, AUC, and Macro-F1 by 0.4, 0.3, and 2.4 percentage points, respectively. The relatively large improvement in Macro-F1 is consistent with the motivation of MFC, which models intra-grade phenotypic variation using multiple feature centers and is intended to improve the discrimination of heterogeneous and borderline DR samples.

Although the incremental experiment characterizes how model performance evolves during the progressive construction of the framework, it does not fully isolate component effects. To provide more controlled comparisons, we therefore further conduct a dependency-aware ablation study, as summarized in Table 9. Starting from the complete configuration, TG-CLM, ODA, and MFC are individually removed while retaining all other compatible components. Because ODA is undefined without the ordinal probability distribution produced by CORN, the contribution of CORN is evaluated through a matched comparison in which ODA is disabled in both configurations. Specifically, TG-CLM+CORN+MFC is compared with TG-CLM+MFC.

As shown in Table 9, removing TG-CLM from the complete framework reduces ACC from 86.3% to 85.4%, AUC from 90.6% to 90.2%, and Macro-F1 from 70.9% to 69.0%. The corresponding decreases of 0.9, 0.4, and 1.9 percentage points indicate that lesion-specific textual guidance remains beneficial even when the ordinal and multi-center constraints are retained. This result is consistent with the role of TG-CLM in extracting lesion-aware representations beyond global image–text semantic alignment.

The effect of ODA can be examined more directly by comparing the full model with the configuration in which ODA is removed while TG-CLM, CORN, and MFC are kept unchanged. Without ODA, ACC, AUC, and Macro-F1 decrease to 86.1%, 90.5%, and 70.1%, respectively. Thus, under the complete configuration, introducing ODA improves the three metrics by 0.2, 0.1, and 0.8 percentage points. This observation differs from the intermediate result in Table 8, where adding ODA before MFC slightly decreases AUC and Macro-F1. Taken together, the two comparisons indicate that the effect of ODA is configuration dependent rather than uniformly positive. In particular, the improved performance observed when both ODA and MFC are present suggests a complementary interaction between ordinal prediction alignment and intra-grade feature regularization.

Removing MFC from the complete model yields ACC, AUC, and Macro-F1 scores of 85.9%, 90.3%, and 68.5%, respectively. Compared with the complete model, the decreases are 0.4, 0.3, and 2.4 percentage points, with the largest degradation observed in Macro-F1. This result suggests that modeling multiple phenotypic centers within each severity grade is particularly helpful for balanced recognition of heterogeneous DR categories.

Because ODA cannot be retained when CORN is removed, directly comparing the full model with a configuration without CORN would simultaneously remove two ordinal components and would therefore confound their individual effects. We instead evaluate CORN by comparing TG-CLM+CORN+MFC (the “w/o ODA” configuration) with TG-CLM+MFC (the “w/o CORN and ODA” configuration), while ODA is disabled in both cases. Introducing CORN increases ACC from 85.2% to 86.1%, AUC from 90.2% to 90.5%, and Macro-F1 from 69.4% to 70.1%, corresponding to improvements of 0.9, 0.3, and 0.7 percentage points, respectively. This matched comparison provides additional evidence that explicit ordinal supervision contributes useful information beyond lesion-aware feature mining and multi-center regularization.

Overall, the incremental and dependency-aware ablation experiments provide complementary evidence regarding the proposed components. The incremental study characterizes how performance changes as the framework is progressively constructed, whereas the dependency-aware study provides more controlled comparisons under compatible model configurations. Importantly, the results do not indicate that every component yields uniform improvements under every intermediate configuration. In particular, ODA introduces a metric-dependent trade-off when added before MFC. Nevertheless, under the complete framework, removing TG-CLM, ODA, or MFC leads to performance degradation, while the matched comparison for CORN also shows improvements across all three metrics. These findings suggest that the final performance gain arises from both the contributions of individual components and their interactions within the joint framework.

#### 4.5.2. Analysis of Global and Lesion-Specific Text Prompts

**Figure 10:**
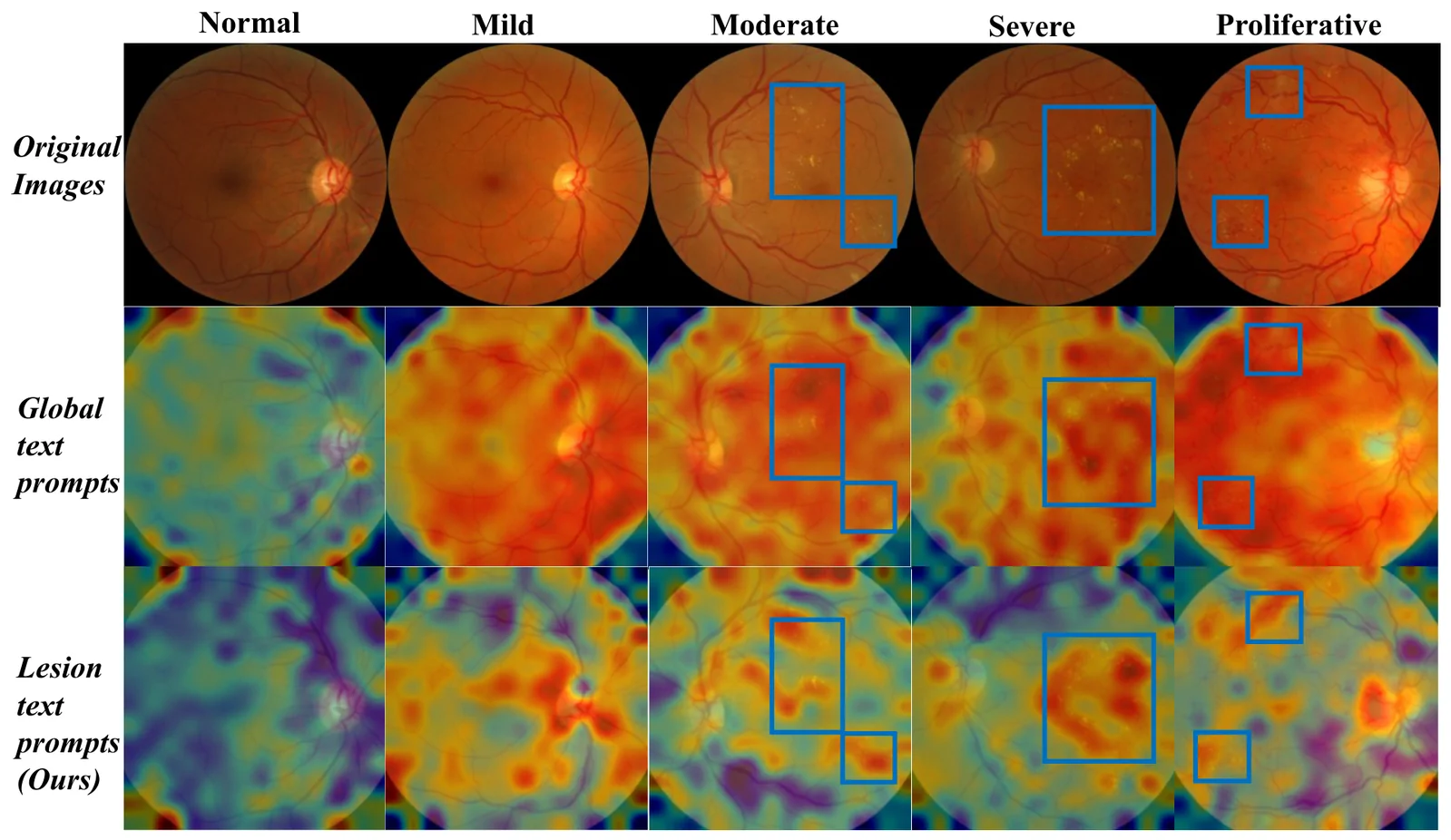
Visualization comparison of lesion-aware attention maps across different diabetic retinopathy grades. The first row shows the original fundus images, the second row presents the attention maps generated using global text prompts, and the third row shows the results produced by lesion-specific text prompts, i.e., our method. The red regions indicate the model’s attention to lesion areas. Blue boxes indicate representative lesion regions.

To investigate the effect of text prompt design in the proposed text-guided cross-layer Lesion Mining (TG-CLM) module, we compare two prompt settings: global text prompts and lesion-specific text prompts. The quantitative experimental results on the validation set are reported in Table 10, and the corresponding attention visualizations are shown in Figure 10.

As shown in Table 10, lesion-specific text prompts achieve better validation performance than global text prompts, with an ACC of 86.3%, an AUC of 92.3%, and a Macro-F1 score of 74.9%. Compared with global prompts, lesion-specific prompts improve ACC, AUC, and Macro-F1 by 0.7, 0.2, and 1.4 percentage points, respectively. The larger improvement in Macro-F1 suggests that lesion-specific prompts are particularly helpful for improving class-balanced recognition across different DR grades. Based on this validation-set comparison, lesion-specific prompts were selected for the final model configuration.

The performance difference can be explained by the different semantic granularity of the two prompt types. Global prompts mainly describe image-level disease categories, such as normal, mild DR, moderate DR, severe DR, and proliferative DR. Although they are useful for global image–text matching, they do not explicitly indicate the pathological evidence associated with DR progression. As a result, the model may still rely on coarse retinal appearance or non-lesion background cues. In contrast, lesion-specific prompts describe clinically relevant findings, including microaneurysms, hard exudates, intraretinal hemorrhages, and neovascularization. These prompts provide more direct semantic guidance for patch-level lesion mining and are therefore better aligned with the objective of TG-CLM.

Figure 10 provides a qualitative comparison of the spatial responses produced by the two prompt settings. Response maps generated using global severity prompts tend to be relatively diffuse and may respond to broad retinal structures, including the optic disc, vessels, and illumination-related regions. In contrast, lesion-specific prompts produce more concentrated responses around several visually identifiable pathological regions, particularly in moderate, severe, and proliferative DR cases. For mild DR, where pathological signs are typically small and sparse, the lesion-specific setting also produces more localized responses around representative abnormalities.

These visual examples qualitatively suggest that lesion-specific textual concepts provide more targeted semantic guidance than global severity descriptions. However, TG-CLM is optimized for image-level DR grading rather than supervised lesion segmentation, and the response maps are not lesion-type-specific binary predictions. Therefore, the visualizations should be interpreted as qualitative evidence of lesion-aware feature mining rather than as a quantitative assessment of pixel-level localization accuracy.

Overall, these results indicate that lesion-specific prompts are more suitable than global prompts for guiding TG-CLM. They improve quantitative performance, especially Macro-F1, and generate attention responses that are more consistent with DR-related pathological regions. This supports the use of lesion-specific textual semantics to bridge clinical lesion descriptions and local visual evidence in five-level DR grading.

#### 4.5.3. Analysis of the Number of Centers in Multi-Center Feature Constraints

**Figure 11:**
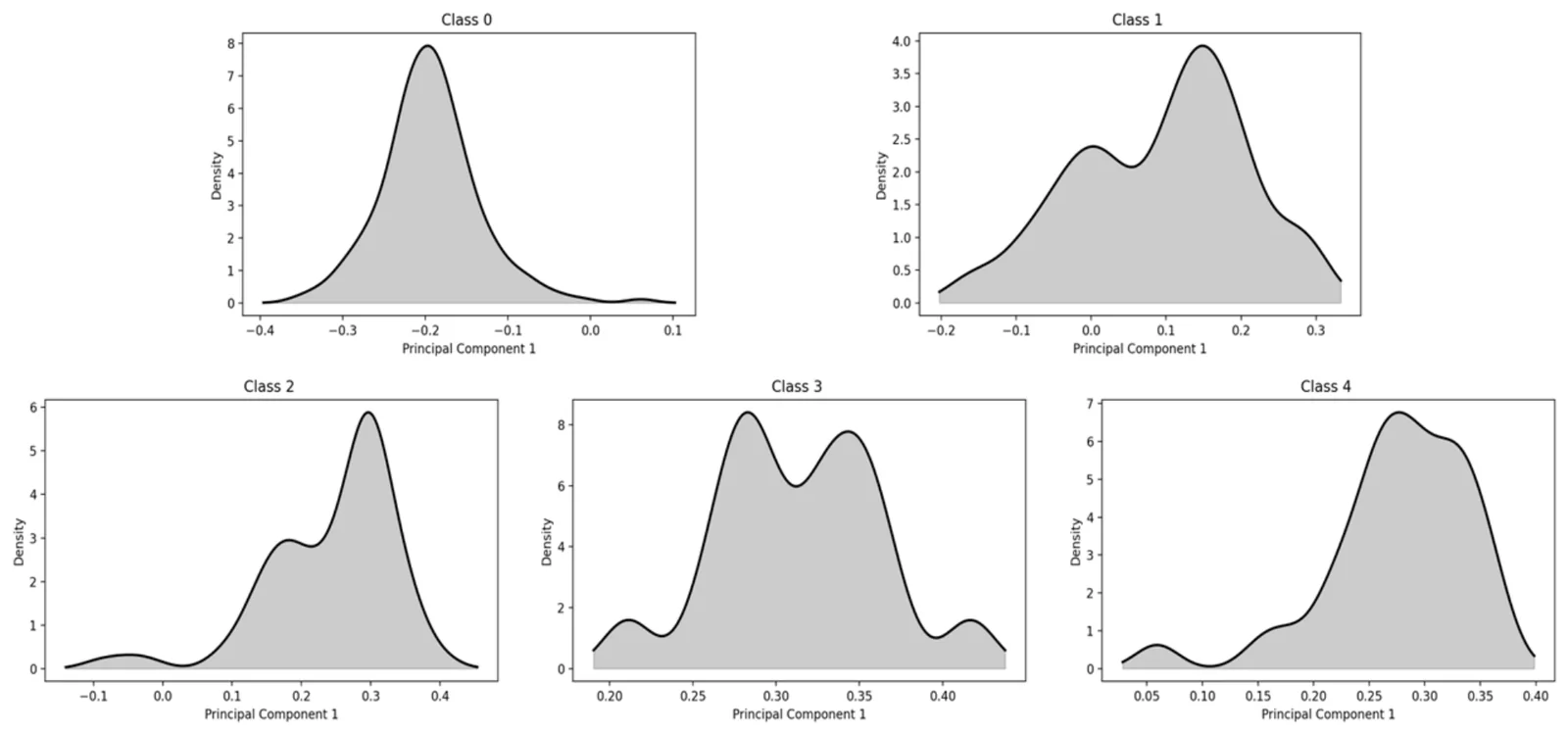
Class-wise feature density distributions after projection onto the first principal component. Different DR grades exhibit different distribution complexities. The normal class shows a relatively compact distribution, while abnormal DR grades present broader or multi-modal patterns, supporting the use of multi-center feature constraints.

To analyze the influence of center configuration in MFC, we first visualize the class-wise feature distributions in Figure 11. The features are projected onto the first principal component, and their density distributions are plotted for each DR grade. As shown in Figure 11, Class 0 exhibits a relatively compact distribution, while the abnormal DR classes show broader or multi-modal patterns. This indicates that normal samples are more visually homogeneous, whereas DR samples within the same grade may contain different lesion combinations and phenotypic appearances.

Based on this observation, we quantitatively evaluate the classification performance of multiple center configuration schemes for multi-center feature constraints, with the detailed results summarized in Table 11. In this table, the notation $\left[n_{0},n_{1},n_{2},n_{3},n_{4}\right]$ represents the number of feature centers allocated to the five DR severity grades, respectively. The uniform single-center configuration [1,1,1,1,1] for all DR grades is adopted as the baseline scheme, which achieves an ACC of 84.7%, an AUC of 92.1%, and a Macro-F1 score of 69.9%. This baseline provides a reliable benchmark for exploring the optimal multi-center allocation strategy targeted at DR classification tasks. Compared with the baseline, the uniform multi-center configuration [1,2,2,2,2], which adds an additional center to all abnormal DR grades, yields limited performance improvement. This configuration only delivers a slight increase of 0.8% in ACC and 0.5% in Macro-F1, while the AUC marginally drops to 91.9%. This phenomenon indicates that blindly increasing the number of feature centers for all abnormal categories cannot effectively boost model performance. The center allocation strategy should be dynamically adjusted according to the intrinsic feature distribution complexity and lesion characteristic differences of each DR grade, rather than adopting a unified allocation rule for all categories.

In line with our core hypothesis, targeted allocation of more feature centers to DR grades with complex feature manifolds and severe lesion features can significantly optimize classification performance. Among all evaluated configuration schemes, the [1,2,2,3,3] setting that increases center quantities for high-grade DR lesions achieves the highest AUC value of 92.7%, demonstrating excellent feature discrimination capability for DR positive and negative samples. On this basis, further optimizing the center budget for moderate and severe DR grades to form the [1,2,3,3,4] configuration enables the model to obtain the optimal comprehensive classification performance. Specifically, this configuration achieves the highest ACC of 86.3% and the best Macro-F1 score of 74.9% across all schemes. Although the [1,2,3,3,4] configuration has a slight AUC decrease of 0.4% compared with the [1,2,2,3,3] scheme with peak AUC, it achieves a substantial improvement in overall classification accuracy and macro-averaged F1 score. This tailored center allocation strategy fully adapts to the feature complexity differences of different DR severity levels, effectively balances the recognition performance of mild, moderate, severe and proliferative DR lesions, and ultimately obtains more stable and superior comprehensive classification results for DR grading tasks.

Therefore, [1,2,3,3,4] is adopted as the final center configuration. This setting is consistent with the feature distributions in Figure 11: fewer centers are sufficient for the compact normal class, while more centers are used for abnormal DR grades with higher intra-grade heterogeneity. It offers a better balance between representation capacity, center stability, and class-balanced performance.

## 5. Discussion

Diabetic retinopathy (DR) grading is a clinically important yet challenging task because disease severity is determined not only by the presence of retinal lesions, but also by subtle differences in their types, quantities, spatial distributions, and progression patterns. Although CNN- and Transformer-based methods have achieved promising performance, they still rely primarily on visual information and often treat the five DR grades as independent categories rather than ordered stages of disease progression. These limitations may lead to insufficient sensitivity to subtle lesions, confusion between adjacent grades, and reduced robustness across datasets acquired under different imaging conditions.

To address these challenges, we propose a text-guided lesion mining vision–language ordinal classification framework for DR grading. TG-CLM uses lesion-specific prompts and normal-tissue descriptions to guide multi-level visual features toward retinal regions relevant to DR grading. A conditional ordinal regression branch and Ordinal Distribution Alignment are introduced to model the ordered progression of DR severity. Meanwhile, MFC employs grade-adaptive phenotypic centers to capture intra-class variations arising from different lesion combinations. Together, these modules enhance lesion-aware representation, ordinal consistency, and feature discriminability.

At APTOS 2019, the proposed method achieves higher ACC, AUC, and Macro-F1 than the RetiZero baseline. Moreover, the repeated-seed statistical analysis shows that the improvements over RetiZero, CLIP-DR, and AOR-DR are statistically significant for all three classification metrics (p<0.05). The ordinal evaluation further shows a higher QWK, lower MAE, higher within-one-grade accuracy, and fewer long-distance grading errors, which is consistent with better preservation of the ordered structure of DR severity on the current test set. The incremental and dependency-aware ablation studies further suggest that the final performance gain arises from both the contributions of individual components and their interactions within the complete framework. In particular, the effect of ODA is configuration dependent rather than uniformly positive across all intermediate settings.

The prompt comparison and visualization results qualitatively suggest that lesion-specific prompts provide more focused semantic guidance toward clinically relevant pathological regions than global category prompts. This may be particularly useful for distinguishing moderate, severe, and proliferative DR, where multiple lesion types can coexist within the same fundus image. However, the response maps are continuous semantic-response maps used for image-level grading rather than lesion-type-specific segmentation masks and therefore should not be interpreted as quantitative evidence of accurate pixel-level lesion localization.

Under the standardized GDRNet leave-one-domain-out benchmark, the proposed method achieves the highest reported average accuracy across six public DR datasets, suggesting its potential for cross-dataset generalization. However, the proposed method does not achieve the highest average performance for all metrics, and the compared approaches retain architecture-specific pretrained encoders. Therefore, these results should be interpreted within the adopted standardized benchmark rather than as evidence of backbone-independent or comprehensive superiority. Several limitations remain, including the absence of quantitative lesion-localization analysis, referable-DR sensitivity and specificity, predictive-calibration assessment, and standardized computational-complexity profiling. Future work will address these issues and investigate stronger domain-generalization strategies.

## 6. Conclusions

In this paper, we propose a text-guided lesion mining vision–language ordinal classification framework for diabetic retinopathy grading that integrates lesion-aware feature mining, ordinal severity modeling, and grade-adaptive phenotypic feature representation. TG-CLM exploits semantic-response differences between normal-tissue and lesion-related textual prompts to guide multi-level visual features toward regions relevant to DR grading, while conditional ordinal regression and ODA explicitly model the ordered progression of DR severity. MFC further employs grade-adaptive phenotypic centers to represent intra-grade variations arising from different lesion combinations. On APTOS 2019, the proposed framework achieves higher ACC, AUC, and Macro-F1 than the controlled baselines. The repeated-seed statistical analysis further shows that the improvements over RetiZero, CLIP-DR, and AOR-DR are statistically significant across all three classification metrics (p<0.05). The proposed method also achieves improved ordinal metrics, including higher QWK, lower MAE, higher within-one-grade accuracy, and fewer long-distance grading errors. The ablation studies suggest that the final performance gain arises from both individual component contributions and their interactions within the complete framework. Furthermore, under the standardized GDRNet leave-one-domain-out benchmark, the proposed method achieves the highest reported average accuracy across six public DR datasets, suggesting potential for improved cross-dataset robustness. However, the domain-generalization results should still be interpreted within the adopted standardized benchmark and should not be regarded as evidence of backbone-independent or comprehensive superiority. Future work will focus on quantitative lesion localization, clinically relevant screening metrics, predictive calibration, computational-complexity analysis, and stronger domain-generalization strategies.

## Figures and Tables

**Table 1:** Summary of diabetic retinopathy grading criteria and representative pathological manifestations based on the International Clinical Diabetic Retinopathy Severity Scale [3].

DR Severity Level	Observable Retinal Findings
No DR	Normal retina without visible diabetic retinopathy lesions.
Mild nonproliferative DR	Microaneurysms only.
Moderate nonproliferative DR	Microaneurysms and other signs (dot or blot hemorrhages, hard exudates, cotton-wool spots), but less severe than severe nonproliferative DR
Severe nonproliferative DR	Any one of the following: intraretinal hemorrhages (≥20 in each of 4 quadrants); venous beading (in 2 quadrants); microvascular abnormalities (in 1 quadrant); and no signs of proliferative retinopathy.
Proliferative DR	One or more of the following: neovascularization, vitreous or preretinal hemorrhage.

**Table 2:** Data distribution of DR datasets.

Datasets	Total	Class 0	Class 1	Class 2	Class 3	Class 4
APTOS	3662	1805	370	999	193	295
IDRID	516	169	25	167	93	62
DEEPDR	1980	908	212	398	350	112
MESSIDOR	1744	1017	270	347	75	35
FGADR	1842	101	212	595	647	287
RLDR	1593	166	337	929	99	62

**Table 3:** Class distribution of the APTOS dataset.

Class	Training	Validation	Testing	Total
0	1434	172	199	1805
1	300	40	30	370
2	808	104	87	999
3	154	22	17	193
4	234	28	33	295
Total	2930	366	366	3662

**Table 4:** Classification comparison results on the APTOS 2019 dataset. Results marked with ^†^ were obtained under the unified experimental protocol used in this study and are reported as mean ± standard deviation over five runs with different random seeds. The remaining results are taken from the corresponding published studies.

Methods	ACC	AUC	Macro-F1
CABNet [44]	83.0	86.3	60.9
CLANet [45]	71.8	–	66.7
LECNet [46]	82.6	87.3	62.0
ADCNet [20]	82.3	84.2	62.5
ABDL [7]	71.8	84.3	61.2
SSIT [14]	77.6	79.8	60.9
DMCLNet [47]	80.5	90.9	59.2
AttMamba [48]	83.2	87.9	68.5
CLIP-DR [34] ^†^	82.6 ± 1.6	89.5 ± 0.7	64.2 ± 2.2
AOR-DR [33] ^†^	84.5 ± 0.8	90.2 ± 0.3	68.1 ± 1.2
RetiZero [16] ^†^	83.9 ± 0.9	89.9 ± 0.4	65.4 ± 1.9
Our method	**86.3 ± 1.2**	**90.6 ± 0.5**	**70.9 ± 2.0**

The optimal result is represented in bold.

**Table 5:** Paired statistical comparison between the proposed method and the controlled baseline methods on the APTOS 2019 dataset. Performance differences are calculated as the proposed method minus the corresponding baseline and are reported as mean ± standard deviation over five seed-matched runs.

Comparison	Metric	Mean Difference ± SD	*p*-Value
CLIP-DR [34]	ACC	3.70±1.89	0.012 *
	AUC	1.10±0.75	0.031 *
	Macro-F1	6.70±2.82	0.006 *
AOR-DR [33]	ACC	1.80±1.17	0.026 *
	AUC	0.40±0.32	0.047 *
	Macro-F1	2.80±1.62	0.018 *
RetiZero [16]	ACC	2.40±1.39	0.018 *
	AUC	0.70±0.53	0.041 *
	Macro-F1	5.50±2.59	0.009 *

Asterisk denotes statistically significant differences (*p* < 0.05).

**Table 6:** Ordinal evaluation of AOR-DR, RetiZero, and the proposed method on the independent APTOS 2019 test set (n=366).

Method	QWK ↑	MAE ↓	W1A (%) ↑	Error ≥2 (%) ↓	Error ≥3 (%) ↓
AOR-DR	0.905	0.197	96.72	3.28	0.82
RetiZero	0.895	0.210	96.17	3.83	1.09
Proposed method	**0.923**	**0.172**	**96.99**	**3.01**	**0.55**

The optimal result is represented in bold. The ↑ denotes higher is better, the ↓ denotes lower is better. W1A denotes the proportion of predictions whose absolute grade error is no greater than one. Error ≥2 and Error ≥3 denote the proportions of predictions with absolute grade errors of at least two and at least three grade steps, respectively.

**Table 7:** Comparison with state-of-the-art approaches under the DG test.

Target	APTOS			DeepDR			FGADR			IDRID			Messidor			RLDR			Average		
Metrics	ACC	F1	AUC	ACC	F1	AUC	ACC	F1	AUC	ACC	F1	AUC	ACC	F1	AUC	ACC	F1	AUC	ACC	F1	AUC
DRGen [49]	58.1	40.2	79.9	38.7	34.1	83.0	41.7	24.7	69.4	44.6	37.4	84.7	60.1	40.5	79.0	43.1	37.0	79.5	47.7	37.3	79.3
Mixup [50]	62.6	43.2	75.3	29.0	25.2	75.3	42.3	32.3	66.7	39.0	27.6	78.8	54.7	32.6	76.7	43.6	37.7	76.9	45.2	33.1	75.0
MixStyle [51]	65.8	39.9	79.0	32.9	27.9	76.9	35.8	22.7	71.2	51.4	39.2	83.0	62.2	36.5	75.2	41.1	31.4	75.5	48.2	32.9	76.8
GREEN [52]	53.8	38.9	75.1	28.1	24.9	76.4	41.3	31.5	69.5	41.3	32.2	79.9	52.0	36.8	75.8	34.0	34.4	74.8	41.8	33.1	75.3
CABNet [44]	55.5	39.4	75.8	42.7	31.8	75.2	43.7	34.8	73.2	44.8	37.3	79.2	56.1	34.1	74.2	37.0	35.6	75.8	46.6	35.5	75.6
DDAIG [53]	67.1	41.0	78.0	37.6	32.2	75.6	42.0	33.8	73.6	37.4	27.0	82.1	58.4	35.3	76.6	36.1	27.7	75.6	46.4	32.8	76.9
ATS [54]	56.9	38.3	77.1	36.1	31.6	79.4	46.7	33.4	74.7	41.5	34.9	83.0	64.7	35.8	77.2	37.4	34.9	76.5	47.2	34.8	78.0
Fishr [55]	66.6	43.4	79.2	48.1	34.4	81.1	44.4	34.4	73.3	40.3	27.6	82.7	**65.1**	41.1	76.4	36.8	34.7	77.4	50.2	35.9	78.4
MDLT [56]	57.2	41.5	77.3	39.5	36.2	80.0	45.7	29.0	74.1	44.2	35.4	81.5	58.9	36.9	75.4	37.6	35.0	75.7	47.2	35.7	77.3
GDRNet [43]	66.8	46.0	79.9	53.1	45.3	84.7	45.3	39.4	**80.8**	40.3	35.9	84.0	63.4	**50.9**	**83.2**	45.8	**43.5**	**82.9**	52.5	43.5	82.6
CLIP [15]	-	44.3	76.1	-	42.0	83.6	-	41.1	78.6	-	34.8	83.0	-	39.6	76.7	-	38.8	75.9	-	40.1	78.9
OrdinalCLIP [57]	-	45.7	77.6	-	43.3	85.1	-	37.9	79.3	-	36.2	80.4	-	41.8	77.7	-	39.5	76.6	-	40.7	79.4
CLIP-DR [34]	-	46.3	83.3	-	**45.8**	**89.9**	-	**48.0**	80.7	-	41.9	84.5	-	47.3	79.0	-	41.0	78.9	-	**45.5**	**82.7**
PAF [58]	68.9	-	-	55.0	-	-	**48.9**	-	-	47.1	-	-	61.8	-	-	**46.1**	-	-	54.6	-	-
Our method	**75.3**	**50.7**	**86.3**	**61.0**	41.1	83.8	45.3	35.4	76.7	**64.5**	**49.2**	**86.5**	**65.1**	38.1	75.7	40.2	34.9	81.6	**58.6**	41.6	81.8

The bold and underline items are the best and the second-best results.

**Table 8:** Incremental ablation study on the APTOS 2019 dataset. TG-CLM denotes text-guided cross-layer Lesion Mining, CORN denotes the Conditional Ordinal Regression branch, ODA denotes Ordinal Distribution Alignment, and MFC denotes the Multi-Center Feature Constraint.

Model	TG-CLM	CORN	ODA	MFC	ACC	AUC	Macro-F1
Baseline					83.9	89.9	65.4
Variant A	✔				84.5	90.3	68.9
Variant B	✔	✔			85.7	90.5	69.2
Variant C	✔	✔	✔		85.9	90.3	68.5
Ours	✔	✔	✔	✔	86.3	90.6	70.9

**Table 9:** Dependency-aware controlled ablation study on the APTOS 2019 dataset.

Configuration	TG-CLM	CORN	ODA	MFC	ACC	AUC	Macro-F1
Full model	✔	✔	✔	✔	86.3	90.6	70.9
w/o TG-CLM		✔	✔	✔	85.4	90.2	69.0
w/o ODA	✔	✔		✔	86.1	90.5	70.1
w/o MFC	✔	✔	✔		85.9	90.3	68.5
w/o CORN and ODA	✔			✔	85.2	90.2	69.4

**Table 10:** Comparison of different text prompt designs for lesion mining on the APTOS 2019 dataset.

Methods	ACC	AUC	F1
Global text prompts	85.6	92.1	73.5
Lesion text prompts (ours)	86.3	92.3	74.9

**Table 11:** Effect of Different Center Configurations in Multi-Center Feature Constraints.

Number of Centers	ACC	AUC	F1
1 1 1 1 1	84.7	92.1	69.9
1 2 2 2 2	85.5	91.9	70.4
1 2 2 3 3	86.1	92.7	73.8
1 2 3 3 4	86.3	92.3	74.9

## Data Availability

All datasets utilized in this study are publicly available. Their corresponding references are [36,37,38,39,40,41] in the manuscript.
